# PICK1 and ICA69 Control Insulin Granule Trafficking and Their Deficiencies Lead to Impaired Glucose Tolerance

**DOI:** 10.1371/journal.pbio.1001541

**Published:** 2013-04-23

**Authors:** Mian Cao, Zhuo Mao, Chuen Kam, Nan Xiao, Xiaoxing Cao, Chong Shen, Kenneth K. Y. Cheng, Aimin Xu, Kwong-Man Lee, Liwen Jiang, Jun Xia

**Affiliations:** 1 Division of Life Science, Division of Biomedical Engineering and State Key Laboratory of Molecular Neuroscience, The Hong Kong University of Science and Technology, Hong Kong, China; 2 Department of Medicine and Department of Pharmacology & Pharmacy, University of Hong Kong, Hong Kong, China; 3 Li Ka Shing Institute of Health Sciences, Faculty of Medicine, The Chinese University of Hong Kong, Shatin, Hong Kong, China; 4 School of Life Sciences, The Chinese University of Hong Kong, Shatin, Hong Kong, China; University of Cambridge, United Kingdom

## Abstract

Diabetes is a metabolic disorder characterized by hyperglycemia. Insulin, which is secreted by pancreatic beta cells, is recognized as the critical regulator of blood glucose, but the molecular machinery responsible for insulin trafficking remains poorly defined. In particular, the roles of cytosolic factors that govern the formation and maturation of insulin granules are unclear. Here we report that PICK1 and ICA69, two cytosolic lipid-binding proteins, formed heteromeric BAR-domain complexes that associated with insulin granules at different stages of their maturation. PICK1-ICA69 heteromeric complexes associated with immature secretory granules near the trans-Golgi network (TGN). A brief treatment of Brefeldin A, which blocks vesicle budding from the Golgi, increased the amount of PICK1 and ICA69 at TGN. On the other hand, mature secretory granules were associated with PICK1 only, not ICA69. PICK1 deficiency in mice caused the complete loss of ICA69 and led to increased food and water intake but lower body weight. Glucose tolerance tests demonstrated that these mutant mice had high blood glucose, a consequence of insufficient insulin. Importantly, while the total insulin level was reduced in PICK1-deficient beta cells, proinsulin was increased. Lastly, ICA69 knockout mice also displayed similar phenotype as the mice deficient in PICK1. Together, our results indicate that PICK1 and ICA69 are key regulators of the formation and maturation of insulin granules.

**Author Summary:**

Insulin is a key regulator of blood glucose and insufficient insulin leads to diabetes. Insulin is synthesized as proinsulin, processed in endoplasmic reticulum and Golgi, and eventually packaged into insulin granules, a type of dense core vesicles. Despite its importance, the molecular mechanisms governing the biogenesis and maturation of insulin granules are not fully understood. In this study, we identified two cytosolic proteins, PICK1 and ICA69, as important regulators of insulin granule biogenesis and maturation. Both PICK1 and ICA69 have the banana-shaped BAR domain that can bend the lipid membrane and help the formation of dense core vesicles. We show that without PICK1 or ICA69, insulin granules cannot be properly formed and, as a result, proinsulin cannot be effectively processed into mature insulin. Mice lacking functional PICK1 or ICA69 genes have reduced insulin but increased proinsulin. Consequently, these mice have high levels of glucose, a prominent feature found in diabetes patients. These results add to previous findings that PICK1 is important for the generation of proacrosomal granules found in cells of the testis, and thereby support a wider role for PICK1 and ICA69 in regulating dense core vesicle biogenesis and maturation.

## Abstract 2

PICK1 and ICA69, proteins containing a BAR domain, regulate the biogenesis and maturation of insulin granules in mice.

## Introduction

Diabetes affects hundreds of millions of people worldwide and its incidence is increasing due to changing lifestyles and an aging population [1]. There are two major types of diabetes, defined by the pathogenic process that causes hyperglycemia [2]. In type 1 diabetes, the destruction of insulin-producing beta cells of the pancreas, mainly by autoimmune processes, results in a gross lack of insulin that leads to hyperglycemia. Type 2 diabetes, on the other hand, is the result of both insulin resistance and insulin insufficiency. Insulin, a peptide hormone secreted by pancreatic beta cells, is a key regulator of blood glucose. It is synthesized as proinsulin that is sorted into immature secretory granules (ISGs) in the TGN [3]–[6]. After budding from the TGN, ISGs go through many changes during their conversion to mature secretory granules (MSGs), changes that include the proteolytic cleavage of proinsulin to insulin, the enrichment of secretory contents, and the removal of unwanted contents by further sorting and budding from ISGs. After maturation, a small fraction of MSGs is mobilized and primed on the plasma membrane to become the readily releasable pool that undergoes regulated exocytosis [7]. In addition to releasing mature insulin via MSGs, beta cells also release proinsulin from ISGs and the elevated ratio of secreted proinsulin to insulin found in patients with type 2 diabetes indicates that the maturation of insulin granules is impaired in this form of the disease [8]. Indeed, recent studies increasingly suggest that impaired insulin trafficking is one of the events underlying the pathogenesis of type 2 diabetes [9]–[11]. However, the molecular machinery responsible for insulin trafficking, such as the sorting, budding, and subsequent refinement of insulin granules, has not been fully elucidated.

Protein trafficking is an elaborated cellular process that involves the coordination of different cytosolic factors, membrane and secreted proteins. PICK1 (protein interacting with C-kinase 1) is a PDZ (PSD-95/Dlg/ZO1) domain-containing peripheral membrane protein that is known to regulate the trafficking of membrane proteins, especially of AMPA receptors in the brain [12],[13]. The PDZ domain of PICK1 binds to membrane proteins and this PDZ–dependent interaction is important for the subcellular localization and surface expression of AMPA receptors [14]–[17]. In addition to the PDZ domain, PICK1 contains a BAR (Bin/Amphiphysin/Rvs) domain, which is capable of sensing membrane curvature and facilitating vesicle formation [18]. This combination of PDZ domain and BAR domain enables PICK1 to link its membrane cargos to trafficking vesicles, a feature found to be critical for AMPA receptor trafficking and synaptic plasticity [17],[18].

We recently identified the protein ICA69 (Islet Cell Autoantigen 69 kD) as a major binding partner of PICK1 [19]. ICA69 was first reported as an autoantigen from type 1 diabetes patients [20]. ICA69 contains a BAR domain at its N-terminus, and a C-terminal domain (the ICAC domain) with no apparent homology with other proteins. ICA69's BAR domain could dimerize with PICK1's BAR domain to form a tight heteromeric BAR domain complex [19]. In neurons, a switch from PICK1-ICA69 heterodimers to PICK1-PICK1 homodimers controls the trafficking of AMPA receptors between dendrites and synapses [19]. In INS-1 beta cell lines, ICA69 associates with the Golgi and partially with ISGs [21],[22], and ICA69 can act as a Rab2 effector for the early trafficking and maturation of dense-core vesicles (DCVs) [23],[24]. Overexpression of ICA69 in INS-1 cells reduced insulin secretion [23], while ICA69 knockout (KO) mice were resistant to cyclophosphamide-accelerated diabetes [25].

Here, we report that PICK1 formed heteromeric complexes with ICA69 in pancreatic beta cells. PICK1-ICA69 complexes were restricted to immature insulin granules and were replaced by PICK1-positive but ICA69-negative complexes when insulin granules mature. Deficiency of PICK1 or ICA69 in mice led to diabetes-like phenotypes characterized by glucose intolerance, insufficient insulin release, and elevated proinsulin secretion. Our findings suggest that PICK1-ICA69 complexes regulate the formation and maturation of insulin granules.

## Results

### PICK1 Is Associated with Insulin Granules

**Figure 1:**
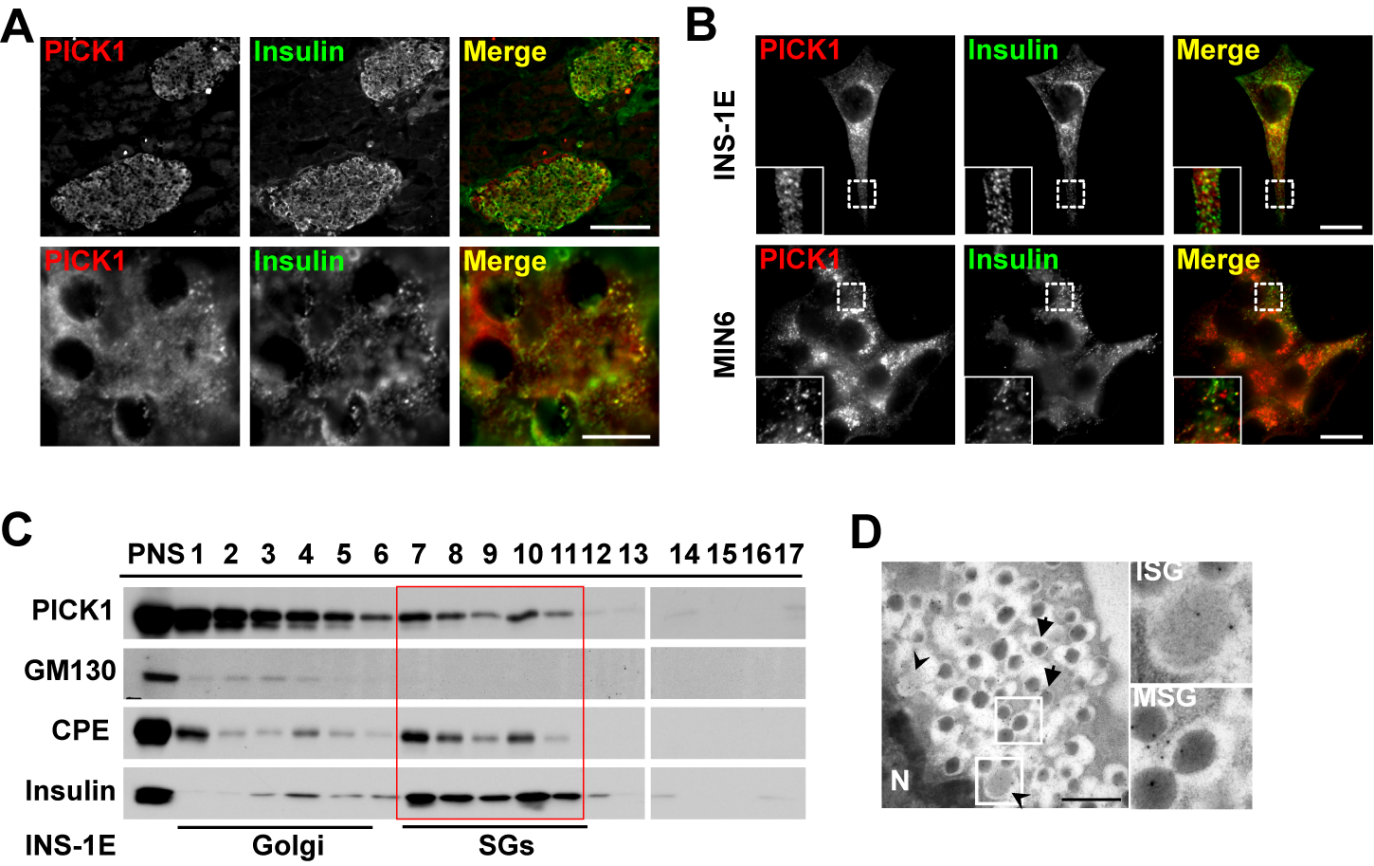
PICK1 is expressed in pancreatic beta cells and partially co-localizes with insulin granules. (A) Upper panel: double staining of PICK1 (red) and insulin (green) on pancreatic cryosection. Scale bar, 100 µm. The lower panel is the magnification of upper panel. PICK1 partially co-localized with insulin positive granules in beta cells. Scale bar, 10 µm. (B) Double staining of PICK1 (red) and insulin (green) on INS-1E cells (upper panel) and MIN6 cells (lower panel). Scale bar, 10 µm. (C) Subcellular fractionation of INS-1E cells. PNS, post-nuclear supernatant; SGs, secretory granules. (D) Immuno-gold labeling of PICK1 on mouse islet beta cell. Arrowheads, ISG (immature secretory granules); arrows, MSG (mature secretory granules); N, nucleus. Scale bar, 1 µm.

To understand the functions of PICK1 in the pancreas, we first examined the localization of PICK1 by immunolabeling sections of mouse pancreas. PICK1 was mainly expressed in islets and specifically in insulin-positive beta cells (Figure 1A, upper panel), with only weak or no expression in the glucagon-positive alpha cells and the somatostatin-positive delta cells (Figure S1A–D). High-magnification images further showed that PICK1 was partially co-localized with insulin granules in the beta cells (Figure 1A, lower panel). To more accurately determine the subcellular localization of PICK1, we also performed immunostaining on cell lines derived from beta cells. In both INS-1E and MIN6 cells, PICK1 was found to partially overlap with insulin granules, although the degree of co-localization was highly variable from cell to cell (Figure 1B). Furthermore, overexpressed mCherry-PICK1 co-localized with the insulin granule marker phogrin-GFP and moved together with it in the cytosol of INS-1E cells (Movie S1). To complement these imaging data with biochemical evidence, we carried out subcellular fractionation: in homogenate of INS-1E cells, PICK1, in addition to being enriched in Golgi fractions, was distributed in insulin fractions marked by Carboxypeptidase E (CPE) and insulin itself (Figure 1C). In addition, transmitted electron microscope (TEM) analysis of immuno-gold labeling on isolated islets from mice confirmed the localization of PICK1 on both immature and mature insulin granules (Figure 1D). Together, these data indicate that PICK1 is associated with insulin granules in beta cells.

### PICK1 Forms Tight Complexes with ICA69 in Beta Cells

**Figure 2:**
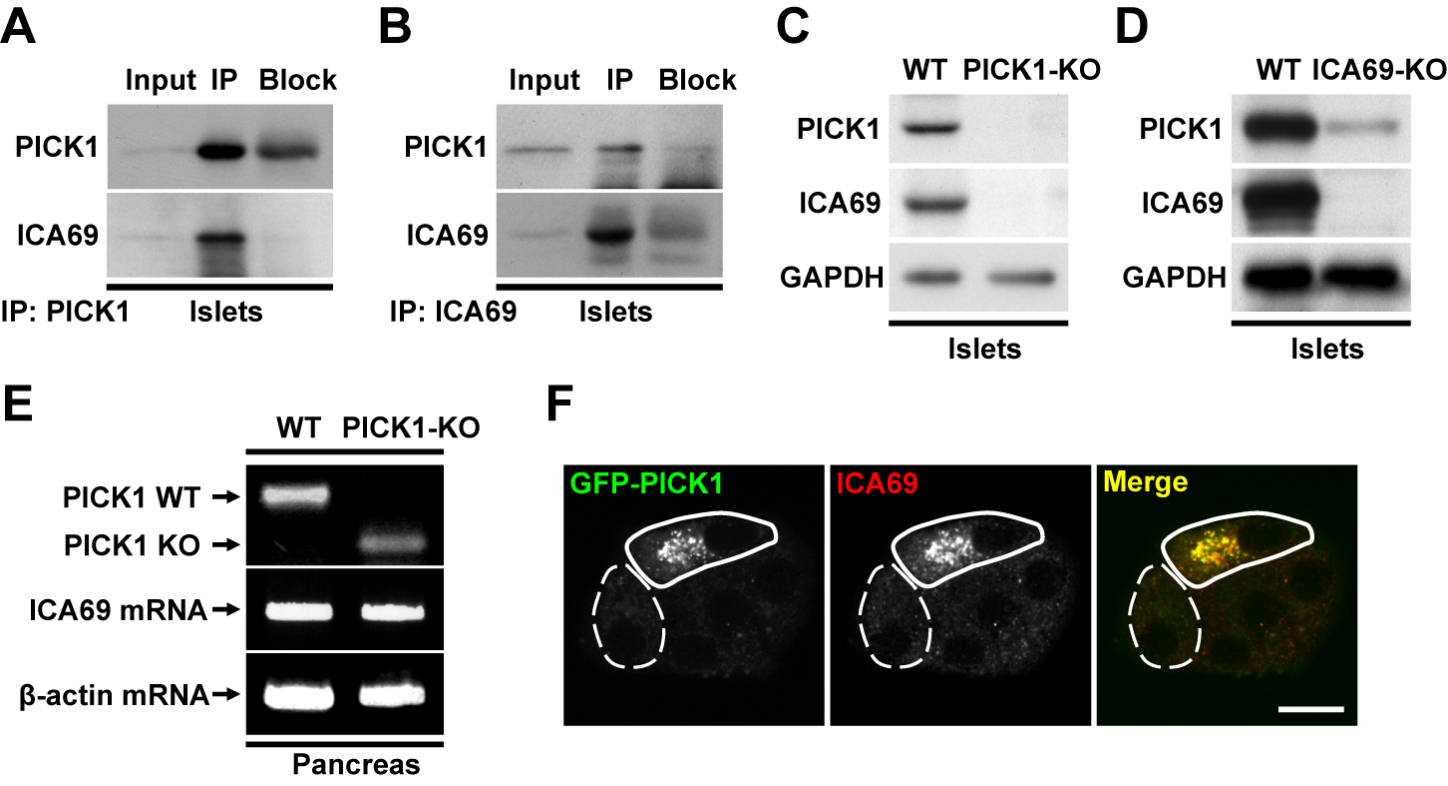
PICK1 and ICA69 form tight complexes in the pancreas. (A) In vivo co-IP from islet extracts using anti-PICK1 antibody. (B) In vivo co-IP using anti-ICA69 antibody. (C) Western blotting of homogenates from WT and PICK1 KO islets. GAPDH served as a loading control. (D) Western blotting of homogenates from WT and ICA69 KO islets. GAPDH served as a loading control. (E) RT-PCR analysis in WT and PICK1 KO pancreas, using β-actin as an internal control. (F) Primary cultured PICK1 KO islet cells were transfected with GFP-PICK1 (green) and stained for ICA69 (red). Scale bar, 10 µm.

PICK1 forms heteromeric BAR domain complexes with ICA69 in the brain [19]. To further explore PICK1's function in pancreatic beta cells, we examined its association with ICA69 using in vivo co-immunoprecipitation (co-IP). When PICK1 was pulled down from extracts of isolated islets, ICA69 was robustly co-immunoprecipitated (Figure 2A). From the other direction, PICK1 was also readily co-immunoprecipitated with ICA69 (Figure 2B). This suggests that PICK1 and ICA69 strongly interact with each other in pancreatic beta cells. Furthermore, we surprisingly found that ICA69 protein was completely missing in PICK1 KO islets (Figure 2C). This lack of ICA69 was not due to down-regulated gene transcription, since RT-PCR analysis showed that ICA69 mRNA levels were similar in the pancreas of PICK1 KO mice and their wild-type (WT) littermates (Figure 2E). To further test PICK1's effect on ICA69 expression, we transfected GFP-PICK1 into PICK1 KO beta cells and found that in these cells the ICA69 protein was restored (Figure 2F). We also measured the level of PICK1 in ICA69 KO mice and found only a small fraction of PICK1 protein still remained in ICA69 KO islets (Figure 2D). These results support our conclusion that PICK1 and ICA69 form tight complexes in the pancreatic beta cells and they mutually depend on each other for normal expression.

### PICK1 and ICA69 Associate with Different Pools of Insulin Granules

**Figure 3:**
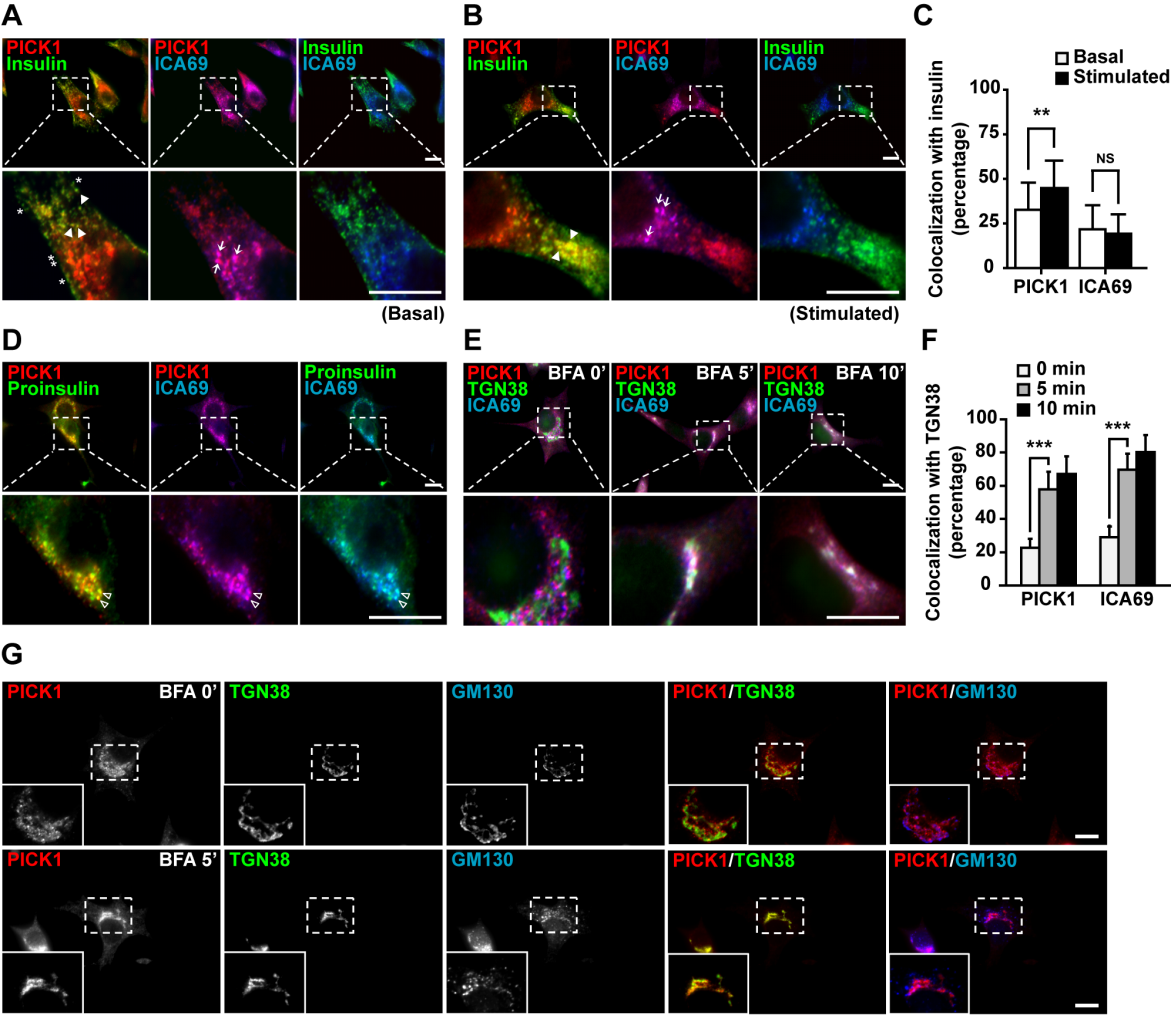
PICK1 and ICA69 associate with insulin granules at three maturation stages. (A) Triple staining of insulin (green), PICK1 (red), and ICA69 (blue) on INS-1E cells. Arrowheads, PICK1-insulin co-clusters without ICA69; arrows, PICK1-ICA69 co-clusters; asterisks, insulin granules without PICK1 and ICA69. Scale bar, 10 µm. (B) Triple staining as in (A) after glucose/KCl-stimulation. Arrows, PICK1-ICA69 co-clusters; arrowheads, PICK1-insulin co-clusters. Scale bar, 10 µm. (C) Co-localization quantification of PICK1 with insulin under different conditions from (A) and (B). Data are represented as mean ± SD; *n* = 31 cells from four independent experiments. **p<0.01. NS, non-significant. (D) Triple staining of proinsulin (green), PICK1 (red), and ICA69 (blue) on INS-1E cells. Empty arrow heads, PICK1-ICA69-proinsulin co-clusters. (E) Triple staining of PICK1 (red), ICA69 (blue), and TGN38 (green) before (0′) and after BFA treatment (5 and 10′). Scale bar, 10 µm. (F) Co-localization quantification of PICK1 or ICA69 with TGN38 from (E). Data are represented as mean ± SD; *n* = 40 cells from three independent experiments. ***p<0.001. (G) Triple staining of PICK1 (red), TGN38 (green), and GM130 (blue) on INS-1E cells without BFA treatment (0′) or with BFA treatment (5′). Scale bar, 10 µm.

To elucidate PICK1/ICA69's functions in beta cells, we examined their subcellular distribution in INS-1E cells relative to insulin granules by immunocytochemistry. PICK1 and ICA69 co-localized at vesicles found mainly around the perinuclear region in INS-1E cells (Figure 3A, center panels; arrows). While the PICK1-positive clusters extended out to the middle cytoplasmic region, where they overlapped with insulin granules (Figure 3A, left panels; arrowheads), the ICA69 signals were confined to the region near nuclei. The insulin-positive granules gradually lost their PICK1 signal as they extended toward the cell periphery (Figure 3A, left panels; asterisks). Thus, the signal for ICA69, unlike that for PICK1, rarely co-localized with insulin (Figure 3A, right panels), indicating that there is no ICA69 on mature granules. To verify the identity of the puncta that were positive for both PICK1 and ICA69, we co-stained them with proinsulin. The PICK1-ICA69 puncta were found to be labeled with proinsulin (Figure 3D, empty arrowheads), indicating that they represent immature insulin secretory granules. Next, to study the dynamics of the association of PICK1/ICA69 with insulin granules, we stimulated INS-1E cells with high glucose and KCl to elicit insulin release. This stimulation increased the signals in the cell periphery for both insulin and PICK1, but not for ICA69. Consequently, granules positive only for PICK1 could be clearly separated from PICK1-ICA69 granules (Figure 3B,C), suggesting that the PICK1 granules are mobilized towards the cell periphery to replenish the depleted readily releasable pool of insulin granules.

Proinsulin is packaged into ISGs in the Golgi. The association of granules positive for PICK1 and ICA69 (P-I granules) with proinsulin prompted us to examine the relationship between the P-I granules and the Golgi. PICK1 and ICA69 were enriched in the TGN region of INS-1E cells, labeled by the TGN marker TGN38 (Figure 3E, left panels). However, they did not fully overlap with each other. Instead, PICK1 and ICA69 signals lined up on the two sides of the TGN38-positive structures (Figure 3E, left lower panels), suggesting that they could be vesicles budding off from the TGN. To test this, we treated INS-1E cells with Brefeldin A (BFA), a drug that blocks vesicle budding from the Golgi [26]. Exposure of INS-1E cells to BFA for 5 min significantly increased the association of both PICK1 and ICA69 with TGN38 (Figure 3D, middle panels), and with treatment for 10 min, PICK1 and ICA69 almost completely overlapped with TGN38 (Figure 3E,F). To distinguish whether the P-I granules were from the trans- or cis-Golgi, we labeled PICK1 together with TGN38 and GM130, which mark the trans- and cis-Golgi, respectively. While PICK1 closely associated with both TGN38 and GM130 before BFA treatment, it co-localized only with TGN38 after exposure to BFA (Figure 3G). These results suggest that PICK1-ICA69 complexes first assemble on the TGN and then bud off to form ISGs.

Here we also examined the relationship of P-I granules with early endosomes and the ER (endoplasmic reticulum). As shown in Figure S2, PICK1 in INS-1E cells did not co-localize with markers for these organelles (EEA1 and Bip/Calnexin for early endosomes and ER, respectively). Because clathrin is well-known for its role in vesicle budding from the TGN and exists on ISGs [27], we double-stained PICK1 with clathrin heavy chain (Clathrin-HC) and clathrin adaptor proteins AP-1, AP-2, and AP-3. PICK1 only slightly overlapped with clathrin and AP-1 at the perinuclear region and showed no co-localization with AP-2 or AP-3 (Figure S3A–D), suggesting that vesicle budding from the TGN mediated by PICK1-ICA69 is clathrin-independent.

### PICK1 KO Mice Have Impaired Glucose Tolerance and Lower Serum Insulin

**Figure 4:**
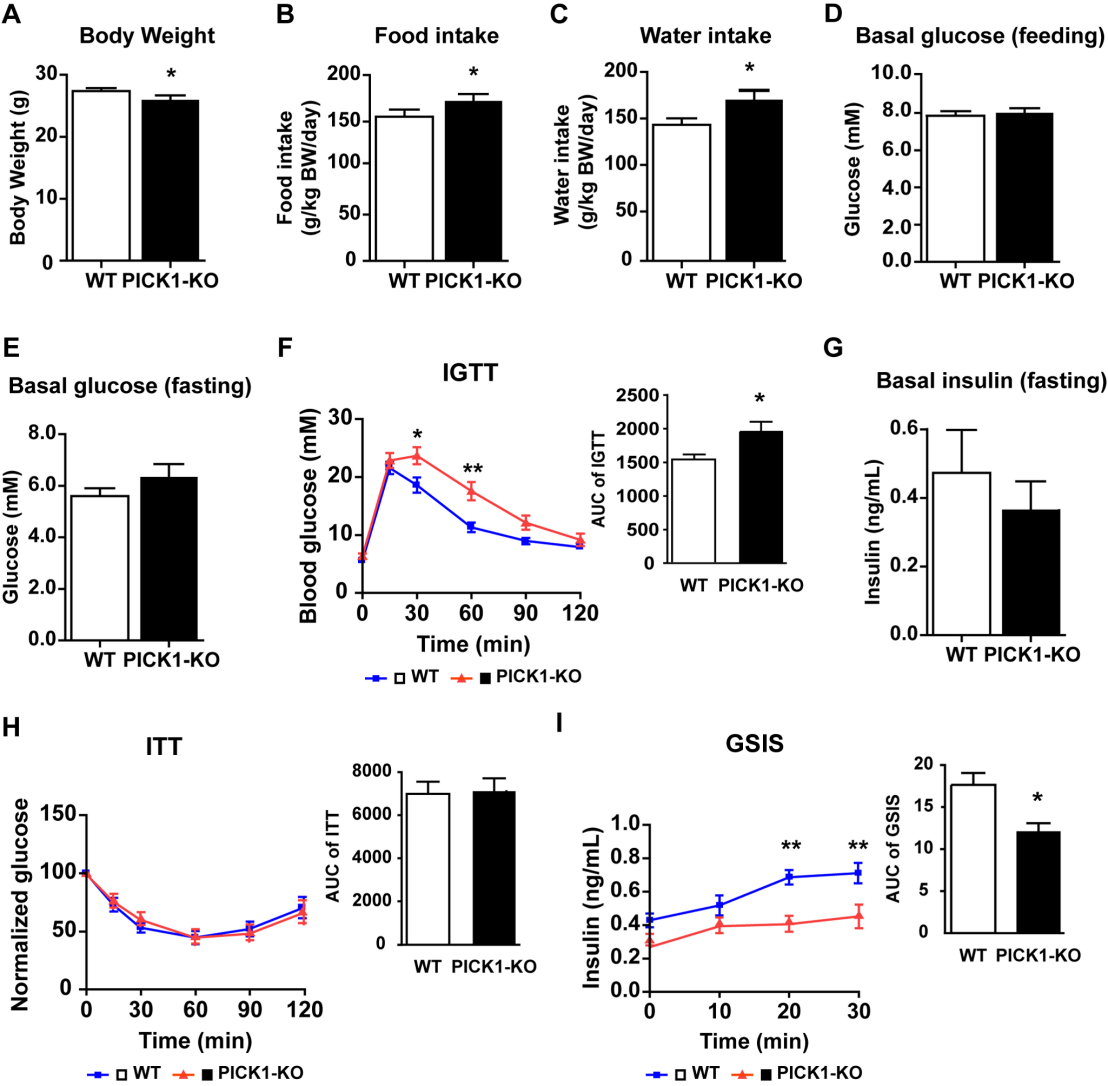
PICK1 KO mice are diabetic due to insulin deficiency. (A) Body weight (*n* = 34, *p<0.05). (B) Daily food intake (*n* = 14, *p<0.05). (C) Daily water intake (*n* = 16, * *p*<0.05). (D) Feeding basal glucose (*n* = 14, *p* = 0.6). (E) Fasting basal glucose (*n* = 16, *p* = 0.17). (F) Intraperitoneal glucose tolerance test (IGTT) and its area under curve (AUC) analysis (*n* = 16, *p<0.05, **p<0.01). (G) Fasting basal insulin (*n* = 20, *p* = 0.47). (H) Insulin tolerance test (ITT) and its AUC analysis. The basal glucose level at 0 min was normalized as 100 (*n* = 20). (I) Glucose-stimulated insulin secretion (GSIS) and its AUC analysis (*n* = 8, *p<0.05, **p<0.01). (A–I) Data are represented as mean ± SEM.

The localization of PICK1-ICA69 complexes in beta cells, coupled with our finding that PICK1 KO mice weighted less than their WT littermates (Figure 4A), led us to examine metabolic parameters in PICK1 KO mice. We first measured the daily food and water intake and found that they were both significantly higher in PICK1 KO mice than in control mice (Figure 4B,C). We then evaluated the body composition and energy expenditure consumption and found these parameters were similar in PICK1 KO and WT mice (Figure S4). The body temperature of PICK1 KO mice, however, showed a slight increase comparing to that of WT mice (Figure S4). PICK1 KO mice also displayed similar levels of nonesterified fatty acids (NEFAs) and cholesterol, but reduced triglyceride and melanocortin (Table S1). We then evaluated the blood glucose in PICK1 KO mice. Compared to controls, PICK1 KO mice showed a small increase in the basal blood glucose level in both feeding and fasting states (Figure 4D,E). This difference became more obvious when we challenged the mice with high glucose, with the PICK1 KO mice now displaying significantly higher blood glucose levels in the intraperitoneal glucose tolerance test (IGTT) (Figure 4F). Because either insulin deficiency or insulin resistance can lead to glucose intolerance, we performed the insulin tolerance test (ITT) to distinguish the two possibilities. No significant difference was found in the insulin sensitivity of peripheral tissues between WT and PICK1 KO mice (Figure 4H), suggesting that the glucose intolerance was not due to insulin resistance. We also checked the insulin downstream signaling in periphery tissues, including liver, muscle, and adipose tissues. Our results found that the phosphorylated Akt and GSK3β, which reflect downstream signaling of insulin, were not much different in PICK1 KO mice compared to controls (Figure S5B–D). This is not surprising given the finding that PICK1 is not expressed in these tissues (Figure S5A). These results further confirm that glucose intolerance is not due to insulin resistance. Thus, to test whether PICK1 KO mice have insulin deficiency, we measured the serum insulin level using enzyme-linked immunosorbent assay (ELISA). The basal insulin level in the fasting state was a little lower in PICK1 KO mice than in WT mice (Figure 4G), but notably, stimulation with glucose significantly increased the serum insulin concentration in WT mice but only slightly in PICK1 KO mice (Figure 4I). These results indicate that PICK1 KO mice have impaired glucose tolerance because of insulin insufficiency.

**Figure 5:**
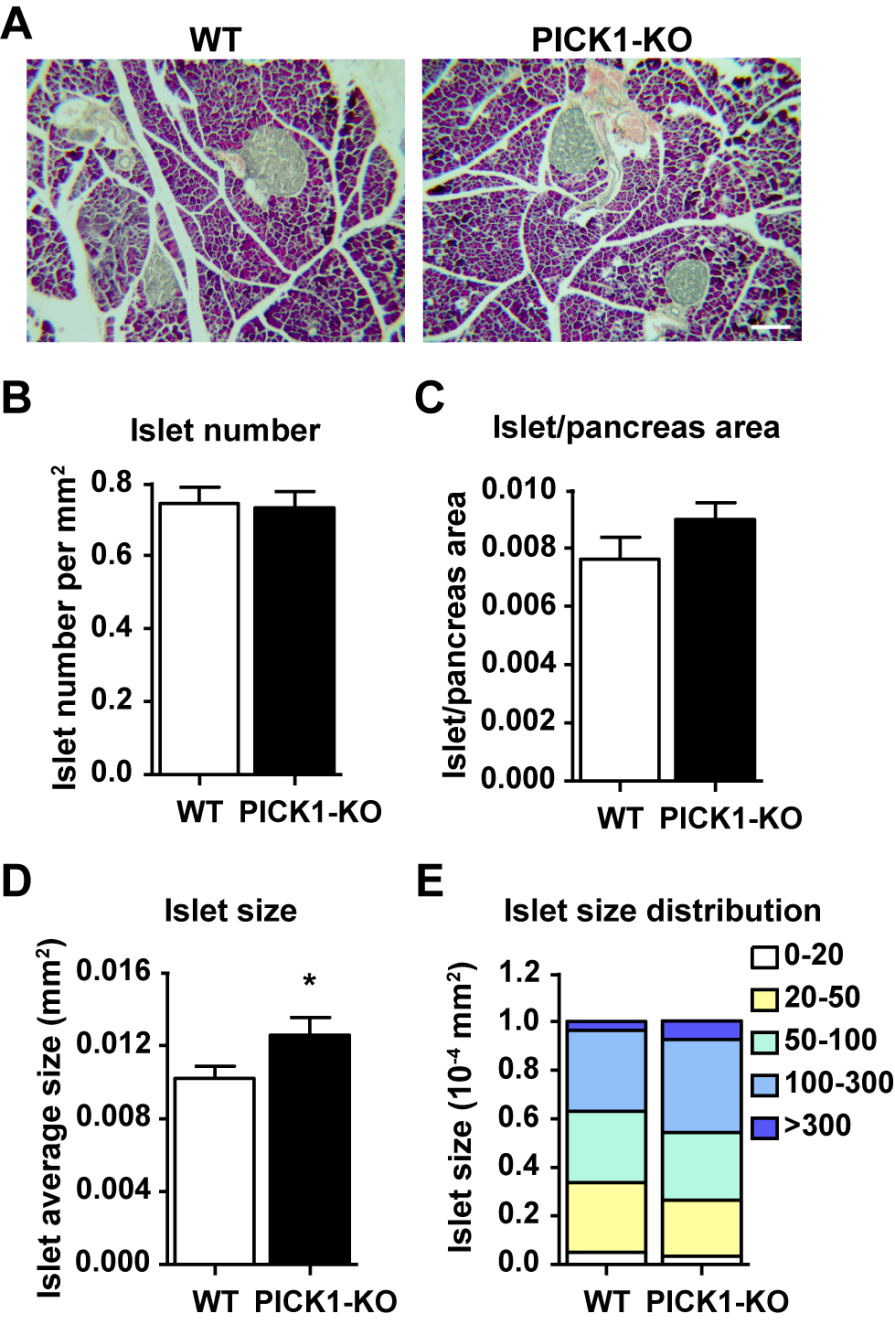
Normal islet structure in PICK1 KO mice. (A) Representative images of H&E staining from WT and PICK1 KO mice pancreas. Scale bar, 100 nm. (B) Quantification of the islet number (*n* = 3, *p* = 0.74). (C) Quantification of islet/pancreas area (*n* = 3, *p* = 0.05). (D) Quantification of islet size (*n* = 3, *p<0.05). (E) Quantification of islet size distribution (*n* = 3). (A–E) Data are represented as mean ± SEM.

To address the cause of insufficient insulin in PICK1 KO mice, we first examined pancreatic islets by Haematoxylin and Eosin (H&E) staining. The morphology and structure of islets from PICK1 KO mice were indistinguishable from that of WT mice (Figure 5A). Quantification from serial sectioning further showed that PICK1 KO and WT mice had similar number of islets (Figure 5B), although the average islet size was increased and the islet/pancreas area was slightly larger in PICK1 KO mice (Figure 5C–E). These results suggested that insulin deficiency in PICK1 KO mice is not due to the reduction of islet mass.

### PICK1 KO Beta Cells Produce Less Insulin but More Proinsulin

**Figure 6:**
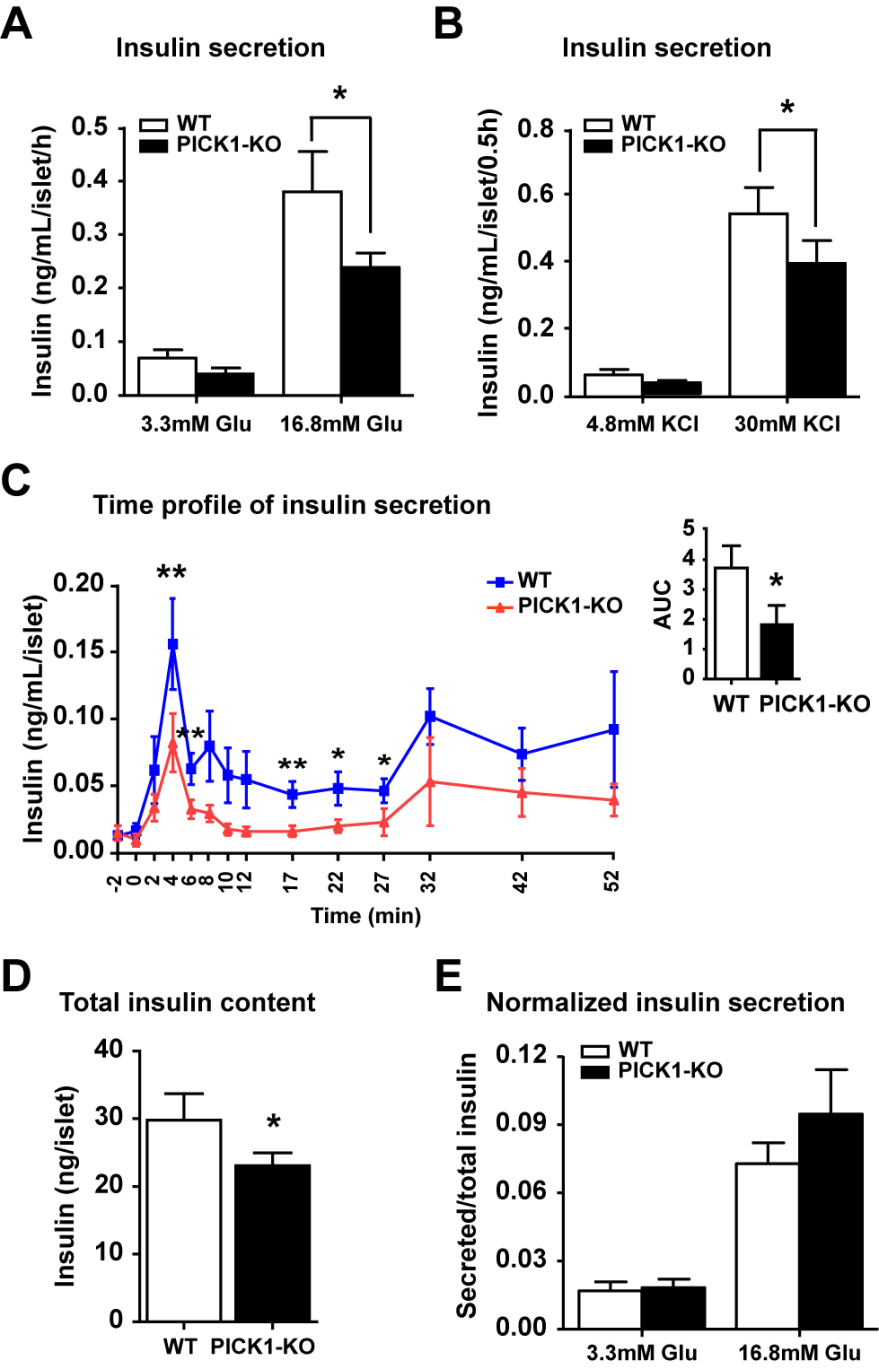
Decreased insulin secretion and total content in PICK1 KO mice. (A) Glucose-stimulated insulin secretion from isolated islets (*n* = 10, *p<0.05). (B) KCl-stimulated insulin secretion from isolated islets (*n* = 9, *p<0.05). (C) Time-course of glucose-stimulated insulin secretion from isolated islets and its AUC analysis (*n* = 10, *p<0.05, **p<0.01). (D) Total insulin content in isolated islets (*n* = 16, *p<0.05). (E) Normalized glucose-stimulated insulin secretion from isolated islets (*n* = 8). (A–E) Data are represented as mean ± SEM.

Since PICK1 is expressed in both pancreatic beta cells and the brain, we tested whether a lower insulin level in PICK1 KO mice is resulted directly from the protein's deficiency in beta cells or from a secondary effect of PICK1 deficiency in the brain. To do this, islets were isolated from PICK1 KO mice and their glucose-stimulated insulin secretion was measured. Basal insulin secretion from PICK1 KO islets was slightly lower than that from control islets, but the glucose-stimulated insulin secretion was significantly reduced compared to WT (Figure 6A). This result indicates that PICK1 deficiency directly affects insulin release from beta cells. Insulin secretion is a process that involves multiple steps, and to ask which of these might be defective in PICK1 KO mice, we used high KCl to stimulate insulin release. High potassium concentration depolarizes the cell membrane and bypasses the need for glucose metabolism followed by the inactivation of ATP-sensitive K^+^ channels. High potassium-induced release of insulin was also much lower in PICK1 KO islets (Figure 6B), indicating that defects in the trafficking of insulin granules, rather than the glucose metabolism, are responsible for the diminished insulin release in PICK1 KO mice.

Insulin secretion occurs by a biphasic process of regulated exocytosis: a rapid first phase is generally believed to represent the exocytosis of insulin granules from the readily releasable pool, while a slow second phase is thought to involve the recruitment of insulin granules from the reserved pool to the readily releasable pool [7]. When we measured the time course of glucose-stimulated insulin secretion from isolated islets, the islets from PICK1 KO mice showed a biphasic pattern of insulin release, like their WT littermates, but the level of insulin release was reduced during the whole process (Figure 6C). This demonstrated that both the first and second phases of insulin secretion are impaired in PICK1 KO beta cells.

**Figure 7:**
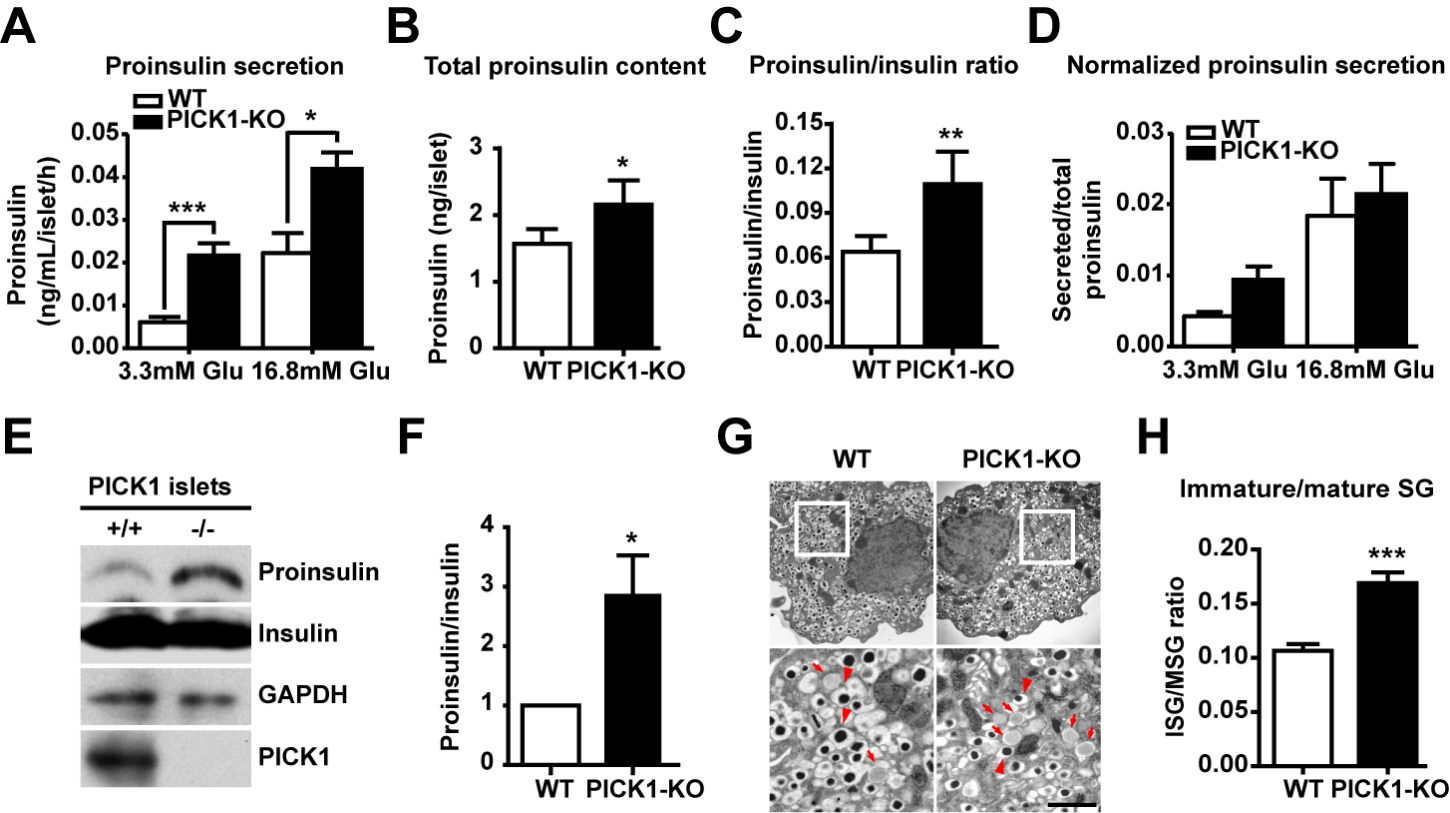
Increased proinsulin secretion in PICK1 KO islets is due to impaired insulin maturation. (A) Glucose-stimulated proinsulin secretion from isolated islets (*n* = 10, *p<0.05, ***p<0.001). (B) Total proinsulin level in isolated islets (*n* = 16, *p<0.05). (C) Proinsulin–insulin ratio in isolated islets (*n* = 16, **p<0.01). (D) Normalized glucose-stimulated proinsulin secretion from isolated islets (*n* = 5). (E) Immunoblotting of proinsulin and insulin from PICK1 KO and wild-type islets. GAPDH served as a loading control. (F) Quantification of proinsulin–insulin ratio in (E) (*n* = 4, *p<0.05). (G) Ultrastructure of PICK1 KO beta cells. Arrows, immature granules; arrowheads, mature granules. Scale bar, 1 µm. (H) Immature/mature secretory granule (SG) ratio in WT and PICK1 KO beta cells (*n* = 37, 48, ***p<0.001). (A–H) Data are represented as mean ± SEM.

The poor secretion of insulin seen above could have been due to abnormal exocytosis or lower intracellular insulin content. We thus measured total insulin in islets, and found that the total insulin level in PICK1 KO islets was significantly reduced relative to control (Figure 6D). After normalizing secreted insulin against total insulin, the percentage of insulin release from PICK1 KO islets became indistinguishable from that of WT islets (Figure 6E). This suggests that lower insulin release from PICK1 KO islets is largely due to the reduced amount of insulin in beta cells. To understand what caused the lower insulin in PICK1 KO beta cells, we measured the proinsulin level in islets. Surprisingly, both glucose-stimulated proinsulin secretion and total proinsulin content were found to be much higher in PICK1 KO islets (Figure 7A,B). And as a consequence, the relative ratio of total proinsulin to insulin was significantly elevated in PICK1 KO than that of WT (Figure 7C). The normalized ratio of secreted proinsulin to total proinsulin, however, was almost the same in WT and PICK1 KO mice, indicating again that the exocytotic machinery is not defective in PICK1 KO mice (Figure 7D). To confirm this result, we performed immunoblotting of proinsulin and insulin using whole islet lysate from PICK1 KO mice. Consistent with ELISA results, we found an increase of proinsulin and a reduction of mature insulin in PICK1 KO islets, comparing to that of wild-type mice (Figure 7E,F). Furthermore, ultrastructural examination of beta cells from WT and PICK1 KO islets by electron microscope (EM) directly revealed that more ISGs were present in PICK1 KO beta cells and that the ISG/MSG ratio was over 50% higher in PICK1 KO beta cells compared to controls (Figure 7G,H). Taken together, these results indicate that the conversion of proinsulin to insulin is impaired in the absence of PICK1 and ICA69.

### Mice Lacking ICA69 Phenocopy PICK1 KO Mice

**Figure 8:**
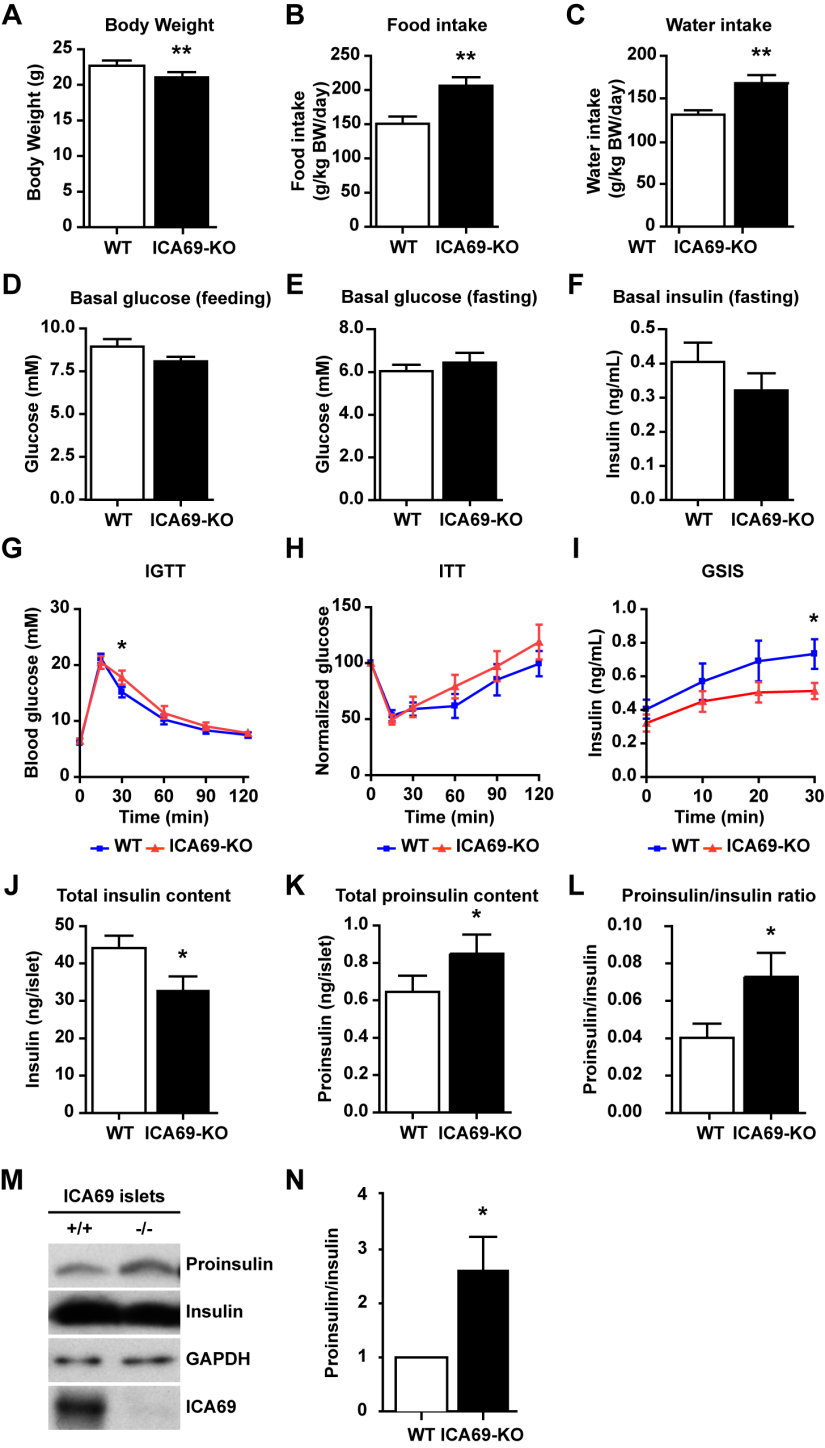
Defective glucose metabolism in ICA69 KO mice. (A) Body weight (*n* = 11, **p<0.01). (B) Daily food intake (*n* = 8, **p<0.01). (C) Daily water intake (*n* = 8, **p<0.01). (D) Feeding basal glucose (*n* = 6, *p* = 0.17). (E) Fasting basal glucose (*n* = 11, *p* = 0.36). (F) Fasting basal insulin (*n* = 8, *p* = 0.21). (G) Intraperitoneal glucose tolerance test (IGTT) (*n* = 11, *p<0.05). (H) Insulin tolerance test (ITT). The basal glucose level at 0 min was normalized as 100 (*n* = 7). (I) Glucose-stimulated insulin secretion (*n* = 8, *p<0.05). (J) Total insulin level in isolated islets (*n* = 5, *p<0.05). (K) Total proinsulin level in isolated islets (*n* = 12, *p<0.05). (L) Proinsulin/insulin ratio in isolated islets (*n* = 6, *p<0.05). (M) Immunoblotting of proinsulin and insulin in ICA69 group islets. GAPDH served as a loading control. (N) Quantification of proinsulin/insulin ratio in (M) (*n* = 4, *p<0.05). (A–N) Data are represented as mean ± SEM.

PICK1 forms tight complexes with ICA69 and PICK1 KO mice lacked ICA69 in beta cells. To further elucidate ICA69's involvement in regulating glucose homeostasis together with PICK1, we examined the phenotype of ICA69 KO mice. ICA69 KO mice in the nonobese diabetic genetic background still develop diabetes, but the disease could not be accelerated by cyclophosphamide [25]. To compare ICA69 KO with PICK1 KO mice, we extensively backcrossed (>10 generations) ICA69 KO mice with C57BL/6 mice to obtain ICA69 KO mice with the same genetic background as PICK1 KO mice. These ICA69 KO mice displayed similar glucose metabolic defects as the PICK1 KO mice, with decreased body weight, increased food/water intake, and impaired glucose tolerance due to insulin deficiency (Figure 8A–I). Isolated islets from ICA69 KO mice also showed reduced insulin and elevated proinsulin content (Figure 8J–N), suggesting that the deficiency of ICA69, like that of PICK1, hindered insulin's maturation. These similarities between the PICK1 KO and ICA69 KO mice, coupled with the other results of this study, strongly support the notion that PICK1 and ICA69 form tight complexes to cooperatively regulate the trafficking of insulin granules.

## Discussion

**Figure 9:**
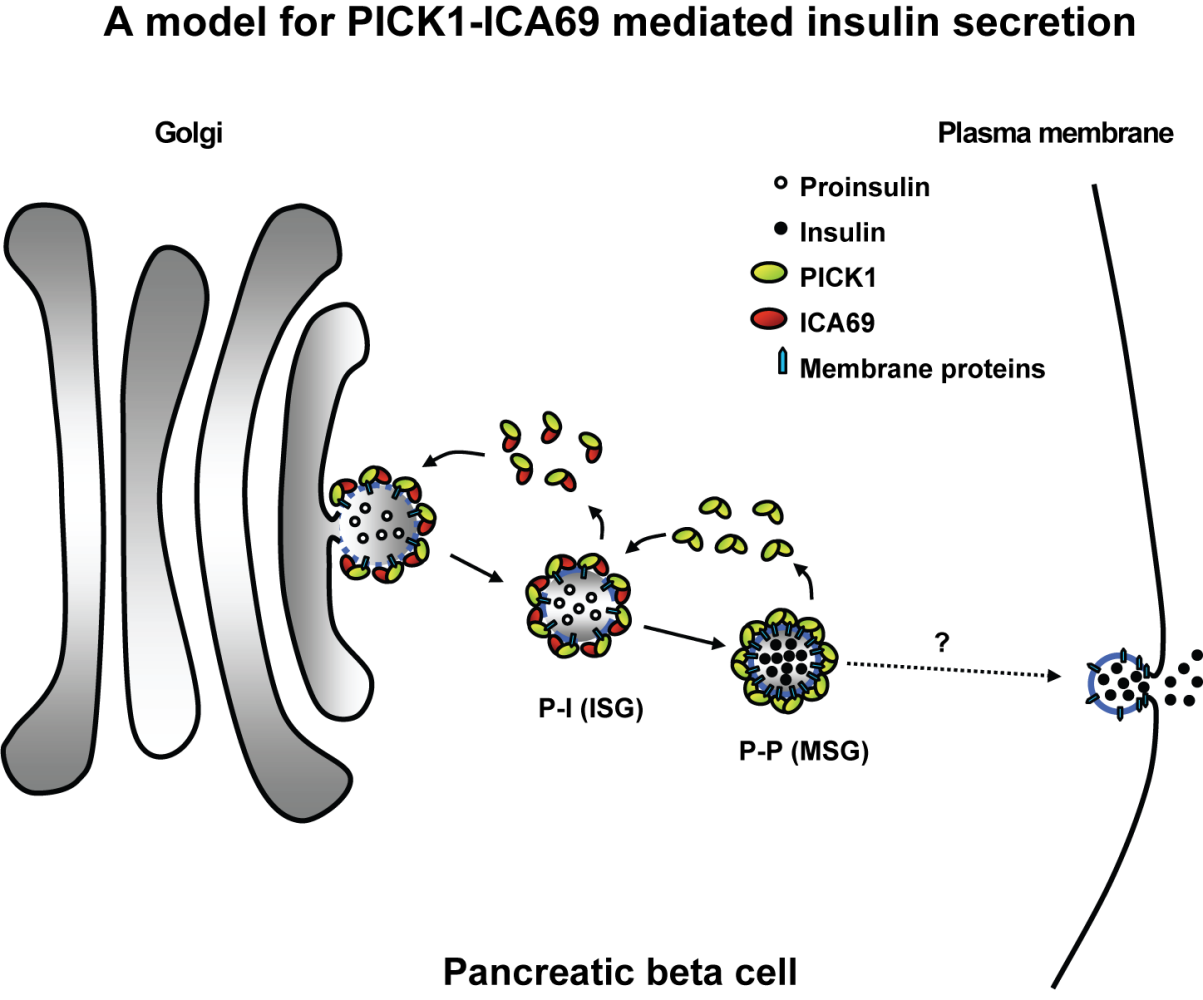
A model illustrating the roles of PICK1 and ICA69 in insulin granule trafficking. In this proposed model, PICK1 and ICA69 form heteromeric BAR domain complexes that are capable of sensing membrane curvatures and phospholipids on the membranes of the TGN. In addition, the PDZ domain of PICK1 may bind to membrane proteins on the insulin granules. This aids the formation of proinsulin immature secretory granules (ISGs) from the TGN with both PICK1 and ICA69 (P-I granules). The P-I granules gradually lose ICA69 as they mature and become PICK1 only granules (P-P granules). With more PDZ domains, the PICK1 homomeric complexes can bind to more membrane proteins and enrich secretory cargos in the mature insulin granules.

The present study revealed the PICK1-ICA69 BAR domain complex as a novel molecular machinery that controls the trafficking of insulin granules. Deficiency of this machinery leads to insufficient insulin that may contribute to the pathogenesis of diabetes. At the subcellular level, the PICK1-ICA69 BAR domain complexes dynamically associate with insulin secretory granules. PICK1-ICA69 heteromeric complexes are mainly localized on the immature proinsulin granules, while PICK1-positive but ICA69-negative complexes, which could be PICK1-PICK1 homomeric complexes, associate with mature insulin granules (Figure 9). When insulin granules further mature, PICK1 eventually disappears from the mature insulin granules. Consistent with the distribution of PICK1 and ICA69, defects of these two proteins impair the maturation of insulin, but not the final exocytotic steps of insulin secretion.

PICK1 contains a PDZ domain and a BAR domain. The PDZ domain of PICK1 interacts with membrane proteins such as GluA2. The BAR domains are known to form banana-shaped dimers that sense curved membrane on vesicles [28],[29]. The unique combination of PDZ and BAR domain on PICK1 makes it capable of coupling membrane proteins to trafficking vesicles [13]. The BAR domain of PICK1 can form homomeric complexes and crosslinks the membrane protein that binds to the PDZ domain, as demonstrated in PICK1's capability to cluster GluA2 in a PDZ-dependent manner [14]. ICA69, on the other hand, contains a BAR domain but no PDZ domain. ICA69's BAR domain forms heteromeric complexes with PICK1's BAR domain and prevents the formation of PICK1 homomeric complexes [19]. It is conceivable that the PICK1-ICA69 heterodimer and PICK1-PICK1 homodimer may have different geometry and lipid-binding preferences. As a result, they may be involved in the formation of different trafficking vesicles. Indeed, the transition of the PICK1-ICA69 heteromeric complex to the PICK1-PICK1 homomeric complex in beta cells marks the conversion from proinsulin granules to mature insulin granules. The exit of ICA69 from the complex enables PICK1 to form homomeric complex and increase its PDZ binding slots. This provides a potential mechanism to enrich membrane proteins on insulin granules that are needed for maturation (Figure 9). It should be noted that the PDZ binding partners of PICK1 in beta cells have not been identified and the detailed mechanism controlling the transition from heteromeric complex to homomeric complex remains to be elucidated.

The roles of PICK1 and ICA69 in insulin trafficking provide the basis to understand the glucose intolerance and insulin maturation defects found in PICK1 and ICA69 KO mice. Loss of PICK1 and ICA69 leads to impaired conversion from proinsulin to mature insulin, and consequently high proinsulin and low insulin in the KO mice. Interestingly, elevated ratio of proinsulin to insulin is a feature found in type 2 diabetes patients, and increasing evidence supports that abnormal insulin trafficking is a key event underlying the disease [8]–[11]. This suggests that PICK1 and ICA69 could be involved in the pathogenesis of diabetes, but it remains to be examined whether abnormalities of PICK1 and ICA69 are indeed present in type 2 diabetes, and what exact roles they may play in the disease.

PICK1 and ICA69 KO mice display a subtle but interesting metabolic phenotype characterized by increased food/water intake and reduced body weight. We, however, could not detect significant changes in body composition and the energy expenditure measured by oxygen consumption. It is possible that the small changes in the fat deposition or energy expenditure measurements were obscured by higher experimental variations. This is supported by the slightly elevated body temperature of PICK1 KO mice (Figure S4G). High fat diet induced obesity makes the alterations in energy homeostasis more pronounced. It would be interesting to see what happens to the fat deposition and energy expenditure of PICK1 KO mice under high fat diet treatment in future studies.

PICK1- and ICA69-mediated vesicle trafficking is not limited to insulin granules. Our previous work found that PICK1 is involved in the trafficking of proacrosomal granules, a type of dense core vesicle in the spermatocytes, and PICK1 deficiency in mice leads to abnormal acrosome formation in sperm and male infertility [30]. A study by Holst et al. demonstrated that PICK1 and ICA69 are also important for the trafficking of growth hormone vesicles in the pituitary gland [31]. There are some minor differences among these two studies; notably, Holst et al. reported no difference in food and water intake, while we found a small increase of food and water intake in PICK1 KO mice. In addition, Holst et al. found PICK1 KO mice had a more sensitive response to insulin, while we did not observe any significant difference. This could be due to the difference in genetic background, as the PICK1 KO mice were backcrossed separately in the two labs. Nevertheless, these differences are minor and both of these studies provided clear evidence supporting PICK1's role in DCV trafficking. Together, these findings suggest that PICK1 and ICA69 may represent parts of a common molecular machinery governing the formation and maturation of DCVs and this machinery is conserved in brain, testis, pancreas, and possibly other neuroendocrine tissues.

## Materials and Methods

### Antibodies

The anti-ICA69 rabbit polyclonal antibody was generated against a peptide corresponding to residues 468–480 (IGKTDKEHELLNA) of rat ICA69. The anti-PICK1 guinea pig polyclonal antibody was raised against the C-terminal 100 amino acids of mouse PICK1 (PC100), while anti-PICK1 rabbit polyclonal antibody was against the N-terminal 29 amino acids (PN29) of mouse PICK1 (PN29). The following antibodies were purchased: anti-PICK1 mouse monoclonal antibody from NeuroMab; anti-insulin, anti-glucagon, and anti-β-tubulin mouse monoclonal antibodies from Sigma; anti-insulin guinea-pig polyclonal antibody for immunoblotting from Abcam; anti-proinsulin mouse monoclonal antibody from Abcam (immunostaining) or R&D systems (immunoblotting); anti-somatostatin rabbit polyclonal antibody from Invitrogen; anti-TGN38, anti-GM130, anti-CPE, anti-EEA1, anti-Bip, anti-calnexin, anti-clathrin-HC, anti-α-adaptin, anti-γ-adaptin, and anti-δ-adaptin mouse monoclonal antibodies from BD-Biosciences; and anti-AKT(total), anti-GSK3β(total), and anti-GSK3β(S9) rabbit polyclonal antibodies from Cell Signaling. The anti-AKT(S473) was generated and tested by Prof. Aimin Xu. Secondary antibodies conjugated with horseradish peroxidase (HRP) were purchased from Amersham, those conjugated with Alexa Fluor-488 or -647 were from Molecular Probes, and Rhodamine RedX-linked antibodies were from Jackson Laboratory. [Note: The insulin antibody used in this study (Sigma, I2018, Clone K36aC10) reacts with both insulin and proinsulin. In our hands, this antibody recognized sharp puncta in the cytoplasm, but its labeling was diffuse in the perinuclear and Golgi regions, suggesting that it may better represent signals from mature insulin granules than those from proinsulin. The mouse anti-proinsulin antibody (Abcam, ab8301, Clone 3A1) is specific for proinsulin and does not recognize insulin or C-peptide.]

### Cell Cultures and Transfection

#### INS-1E cell culture and transfection

INS-1E cells (generously donated by Dr. Pierre Maechler, University Medical Center, Geneva, Switzerland) were cultured in a humidified atmosphere containing 5% CO2 in a complete medium composed of RPMI1640 (Invitrogen) supplemented with 10% fetal bovine serum, 1 mM sodium pyruvate, 50 µM 2-mercaptoethanol, 2 mM glutamine, 10 mM HEPES, 100 U/mL penicillin, and 100 µg/mL streptomycin. These cells were transiently transfected using Lipofectamine 2000 reagent (Invitrogen) following the manufacturer's instructions.

#### MIN6 cell culture

MIN6-B1 cells (generously donated by Dr. Philippe Halban, University Medical Center, Geneva, Switzerland) were cultured in DMEM supplemented with 15% fetal bovine serum, 25 mM glucose, 71 µM 2-mercaptoethanol, 2 mM glutamine, 100 U/mL penicillin, and 100 µg/mL streptomycin.

#### Primary pancreatic cell culture and transfection

After isolation and recovery, pancreatic islets were treated with 0.5% Trypsin in PBS for 5 min at 37°C. The dispersed cells were cultured in RPMI 1640 medium and used 5–6 d after plating. Transient transfections were again performed using Lipofectamine 2000 reagent according to the manufacturer's instructions.

### Live Imaging

INS-1E cells were transfected with mCherry-PICK1 and phogrin-GFP on Day 3 after culturing. Imaging was performed at 37°C with 5% CO_2_ 2 d later. Time-lapse images were acquired with a Nikon TE2000E-PFS microscope equipped with a Tokai Hit INU-NI-F1 temperature, humidity, and CO_2_ chamber. Images were acquired at 2-s intervals with 200 ms exposures. Data acquisition and image processing were performed using MetaMorph (Universal Imaging) and Image J software (NIH).

### Co-Immunoprecipitation

About 100 isolated islets were solubilized using 1% Triton X-100 with the protease inhibitor cocktail (Roche) for 30 min at 4°C. Anti-PICK1 or anti-ICA69 sera were pre-incubated with Protein A Sepharose (GE Healthcare) for 1–2 h at 4°C. The clarified supernatant of the islet lysates were then added to the beads. The mixtures were then incubated for 2–3 h at 4°C. After washing, the immunoprecipitates were eluted from the beads and resolved by SDS-PAGE for analysis by Western blotting. For peptide-block controls, sera were pre-incubated with PICK1 N29 peptide or ICA69 C-terminal peptide for 2 h. Anti-PICK1 mouse monoclonal antibody was used for Western blotting to eliminate the IgG background in IP samples.

### Subcellular Fractionation

Four 100-mm dishes of INS-1E cells were washed once with ice-cold PBS and scraped in 5 mL ice-cold PBS with 1 mM phenylmethylsulphonyl fluoride (PMSF). Cells were homogenized in 5 mL Buffer A (0.3 M sucrose, 1 mM EDTA, 1 mM MgSO_4_, 10 mM MES-KOH, pH 6.5) on ice, and the homogenate was centrifuged at 1,000 *g* for 5 min to remove unbroken cells and nuclear debris. The postnuclear supernatant (PNS) was collected and loaded on top of a discontinuous Optiprep (Sigma) gradient composed of five layers (2 ml 30%, 2 ml 23.4%, 2 ml 17.6%, 2 ml 13.2%, 2 ml 8.8%) in a SW40 tube, and the sample was centrifuged at 100,000 *g* for 75 min. About 600 µL for each fraction was collected for Western blotting analyses.

### Western Blotting of PICK1 Pancreatic Tissue and Proinsulin/Insulin

Pancreases were homogenized in a homogenizing buffer (10 mM Tris-Cl, 320 mM sucrose, pH 7.4) to obtain total protein extracts. Protein concentrations were determined by Coomassie assays (Pierce, Rockford, IL). Equal amounts of proteins (∼20 µg/lane) were resolved by SDS-PAGE and analyzed by Western blotting using anti-PICK1, anti-ICA69, and anti-β-tubulin antibodies. To detect proinsulin/insulin, the islet lysates were subjected to the nonreducing Tricine (16%)/Urea (8M) SDS-PAGE and analyzed by Western blotting.

### Genotyping PCR and RT-PCR

Mice were genotyped by PCR using a 3-primer design: 5′ TCACTTGCCAGAGGAGAAAACTG 3′, 5′ AAAAATAGGCGTATCACGAGGC 3′, and 5′ CACTCGCAGCTTGTTCTGATCTG 3′. The WT PCR product is a 400 bp band while the mutant band is 200 bp.

Pancreas from WT and PICK1 KO mice were used for RT-PCR analysis. Pancreatic total RNA was extracted with TRIzol reagent (Invitrogen) as described in the manufacturer's instructions, and cDNAs were prepared by reverse transcription using First-strand cDNA Synthesis kit (Fermentas). PCR was done with Platinum Taq DNA Polymerase (Bioline). The primer pairs for mouse ICA69 were forward 5′AAGGATGACCTCTTGCTGTTGAATG 3′ and reverse 5′ ATAGCGATAGAAACAGGGCCTTGAC 3′. Those for mouse β-actin were forward 5′ TGAGAGGGAAATCGTGCGTG 3′ and reverse 5′ TGCTTGCTGATCCACATCTGC 3′.

### Immunocytochemistry

INS-1E cells, MIN6 cells, or primary cultured islet cells were fixed by 4% paraformaldehyde plus 4% sucrose in PBS for 20 min at room temperature. The cells were then permeabilized by 0.2% Triton X-100 in PBS for 10 min, blocked with 10% normal donkey serum (NDS) in PBS for 1 h, and then sequentially probed for 1 h each with primary antibodies and fluorescent secondary antibodies diluted in 3% NDS (all at room temperature). After washing with PBS, coverslips were mounted with Permafluor (Immunon). For the treatment with high glucose and KCl, INS-1E cells on day 5 were first pre-incubated in 3.3 mM glucose/KRBH for 30 min, and then cells were transferred to 16.8 mM glucose and 70 mM KCl or further incubated in 3.3 mM glucose for 60 min. For the treatment with Brefeldin A (BFA), INS-1E cells were treated with 2 µg/mL BFA (Sigma) or vehicle control of absolute ethanol at various time points as mentioned in the results. For the treatment with high glucose, primary cultured beta cells on day 6 were washed with KRBH buffer once and then incubated in 16.8 mM glucose for 30 min. Cells were examined using a Nikon Eclipse TE2000 inverted fluorescence microscope or Zeiss LSM510 confocal laser scanning microscope under a 60× or 100× Plan Apochromatic oil lens (NA = 1.4).

### Immunohistochemistry

Pancreases were first fixed with 4% paraformaldehyde and 4% sucrose in PBS for at least 4 h at 4°C and then cryo-protected by incubation in gradient sucrose-PBS solutions at 4°C (10% sucrose 1 h, 20% sucrose 1 h, and 30% sucrose overnight). Cryosections of 10 µm thickness were used for immunohistochemical analysis. Sections were rinsed with PBS, permeabilized, and blocked with 10% normal goat serum plus 0.2% Triton X-100 in PBS for 1 h at room temperature and then incubated overnight with primary antibodies at 4°C in a humidified atmosphere. After gently washing thrice with PBS and incubating with fluorescent secondary antibodies for 1 h at room temperature, sections were dehydrated and mounted with Vecta Mount solution (Vector Labs).

### Haematoxylin and Eosin (H&E) Staining and Islet Morphology Analysis

Pancreases were fixed with 4% paraformaldehyde and 4% sucrose in PBS for 24 h at 4°C. After dehydration, samples were embedded in paraffin and serial paraffin sections were obtained at 100-µm intervals from each pancreas. In all, 12 sections (7 µm thick) were used for quantification. Standard H&E staining was performed, and the slides were mounted using Vecta Mount solution. The islet/pancreas area, the islet number/mm^2^, and the average size of islets were determined by the MetaMorph image acquisition software.

### Transmission Electronic Microscopy

TEMs were performed as previously [30]. Briefly, isolated islets were fixed with 2% glutaraldehyde overnight at 4°C, dehydrated, and then embedded in Spurr's resin (Electron Microscopy Science, Hatfield, PA). Ultrathin sections (70 nm) were cut on an ultratome (Leica, Reichert Ultracuts). Spur sections were directly poststained with aqueous uranyl acetate/lead citrate. The observations were performed with a Hitachi H-7650 transmission EM operating at 80 kV, and pictures were taken with a Hitachi AMT –XR 40 CCD camera. The secretory granule size, area, and number were quantified by the Image J software.

For immunolabeling, isolated islets were fixed in 0.25% glutaraldehyde and 1.5% paraformaldehyde in 50 mM Caco buffer. Thereafter, the samples were dehydrated through a graded ethanol series and then embedded in Lowicryl Resin HM20 (Electron Microscopy Science, Hatfield, PA). Ultrathin sections (70 nm) were blocked in 3% BSA for 20 min at room temperature and probed with mouse anti-PICK1 antibody for 3 h. After washing in 1% BSA, sections were exposed for 1 h to anti-mouse IgG secondary antibodies conjugated to 10 nm gold particles (Electron Microscopy Science) and then washed in 1% BSA. The sections were post-stained with aqueous uranyl acetate/lead citrate.

### Analysis of Metabolic Parameters

#### Body weight

The body weights of WT or KO mice were measured at ages between 6 to 8 mo.

#### Food and water intake

Body weights of 6–8-mo-old mice were measured before tests and the 24 h intake of food and water was recorded for 4 d to calculate the average values of consumed food (g) or water (g)/kg body weight/day.

#### Body composition

The body composition was measured by Bruker LF90 MiniSpec analyzer.

#### The energy expenditure measurement

The mice were adapted in the metabolic cages (CLAMS, Columbus Inc.) for 24 h before the measurement. The O_2_ consumption and RER were recorded automatically for 72 h.

### Measurement of Basal Glucose and Insulin Levels

For the fasting condition, 4–6-mo-old mice were starved for 16 h and blood was collected from the tail vein. Basal glucose levels were measured by a glucometer (One Touch Ultra, LifeScan), and basal insulin levels were measured by an Ultra-Sensitive Mouse Insulin ELISA kit (Crystal Chem.). In the feeding condition, mice given a normal diet were tested at 9:00 am and blood glucose levels were measured by the glucometer.

### IGTT

We starved 4–6-mo-old mice for 16 h or overnight before being administered an intraperitoneal injection of glucose (1.5 g/kg body weight). Blood was drawn from the tail vein before and 15, 30, 60, 90, and 120 min after injection to measure glucose levels using the glucometer.

### ITT

We fasted 4–6-mo-old mice for 4–5 h prior to the ITT test. Human regular insulin (0.1 U/mL in saline; Roche) was intraperitoneally injected into each mouse (0.75 U/kg body weight), and blood glucose levels were measured by the glucometer at 0 min (before injection) and 15, 30, 60, 90, and 120 min after injection.

### Glucose-Stimulated Insulin/Proinsulin Secretion Assay

#### Insulin secretion assays on mice

We fasted 10–12-mo-old WT and KO mice for at least 16 h, and followed by injection with glucose (1.5 g/kg body weight) as described for the IGTT assay. Blood (>20 µl) from the tail vein was collected before and 10, 20, and 30 min after injection. The blood was spun at max speed in a tabletop centrifuge for 10 min at 4°C and the supernatant serum was collected. The insulin concentrations in these samples were measured by an insulin ELISA kit using mouse insulin as the standard.

#### Insulin secretion assays on isolated islets

We sacrificed 10–12-mo-old mice by cervical dislocation. For islet isolation, the common bile duct was cannulated and injected with 3 mL cold HBSS medium containing 1 mg/mL collagenase V (Sigma). The distended pancreas was incubated in a water bath at 37°C in 1 mg/mL collagenase V in HBSS for 10–13 min. The islets were separated on Histopaque 1077 (Sigma) density gradients and then hand-picked under a dissecting microscope to ensure that the islet preparation was pure. After overnight culture in RPMI 1640 containing 11 mM glucose, islets of similar size from WT and PICK1 KO mice were handpicked and pre-incubated for 1 h in KRBH buffer containing 3.3 mM glucose. Groups of 15 islets were incubated in 1 mL KRBH buffer containing 3.3 mM glucose for 1 h more and then transferred to 1 mL KRBH buffer containing 16.8 mM glucose for another hour. After incubation, the KRBH buffer was collected and spun down at 500 g for 5 min. The supernatant was stored at −20°C, and the insulin concentration was later measured by an insulin ELISA kit. Total insulin content was extracted by acid-ethanol (70% ethanol +0.18 M HCl) at 4°C for 12 h. For KCl stimulation assays, after the pre-incubation step, 15 islets were incubated in 1 mL KRBH buffer containing 4.8 mM KCl for 30 min and then in 1 mL KRBH buffer containing 30 mM KCl for another 30 min. The supernatant was collected for insulin concentration measurement. For time-course experiment, 15 islets were pre-incubated in KRBH buffer containing 3.3 mM glucose for 1 h. The islets were then incubated in 1 mL KRBH buffer containing 16.8 mM glucose for 2 min. At different time points, the islets were transferred to 1 mL fresh KRBH buffer containing 16.8 mM glucose for 2-min incubation. Supernatants from different time points were collected to measure insulin concentrations and to generate time-course curves.

#### Proinsulin secretion on isolated islets

Isolated islets were treated with glucose as in insulin secretion assays. Secreted and total proinsulin content was measured using rat/mouse proinsulin-specific ELISA kits (Mercodia).

### Statistical Analyses

All data are presented as mean ± SEM, and *p* values are from two-tailed Student's *t* tests. Values of *p*<0.05 were considered as statistically significant.

## Supporting Information

Figure S1 PICK1 is mainly expressed in pancreatic beta cells but not alpha or delta cells. (A) Immunostaining of PICK1 (red) on WT and PICK1 KO pancreatic cryosections. DAPI was used to label nucleus. Guinea pig anti-PICK1 antibody recognizes nonspecific signals at the periphery region of islets (arrows). Scale bar, 100 µm. (B) Double staining of PICK1 (red) and insulin (green) on cultured islet cells. PICK1 is highly expressed in the insulin-positive pancreatic beta cells. Scale bar, 10 µm. (C) Double staining of PICK1 (red) and glucagon (green) on cultured islet cells. PICK1 is weakly expressed in the glucagon-positive pancreatic alpha cells compared with neighboring glucagon-negative cells. Scale bar, 10 µm. (D) Double staining of PICK1 (red) and somatostatin (green) on cultured islet cells. No PICK1 expression could be detected in the somatostatin-positive pancreatic delta cells compared with neighboring somatostatin-negative cells. Scale bar, 10 µm. DAPI was used to label nucleus.
(TIF)

Figure S2 PICK1 vesicles are related to Golgi structures but not early endosomes or ER. Double staining in INS-1E cells for (A) PICK1 (red) and EEA1 (green), (B) PICK1 (red) and Bip (green), and (C) PICK1 (red) and calnexin (green). (A–C) Scale bar, 10 µm.
(TIF)

Figure S3 The relationship between PICK1 and clathrin and its adaptors. Double staining in INS-1E cells of (A) PICK1 (red) and Clathrin-HC (green), (B) PICK1 (red) and γ-adaptin (green), (C) PICK1 (red) and α-adaptin (green), and (D) PICK1 (red) and δ-adaptin (green). Scale bar, 10 µm.
(TIF)

Figure S4 Unaltered body composition and energy expenditure in PICK1 KO mice. (A) Fat mass percentage and (B) lean mass percentage was measured by NMR system (12-wk-old male mice; *n* = 6). (C–D) Oxygen (O_2_) consumption. (E–F) Respiratory exchange rate (RER) was measured using metabolic cages for 48 h. (Light, 7 am–7 pm; Dark, 7 pm–7 am; 12-wk-old male mice, *n* = 6). (G) Body temperature, as measured by anal temperature at 9 am daily (12-wk-old male mice; *n* = 6). (A–G) Data are represented as mean ± SEM, Student's *t* test.
(TIF)

Figure S5 Insulin effects on PICK1 KO target tissues. (A) Western blotting of PICK1 in adipose, muscle, and liver tissue lysates. WT brain tissues were used as a PICK1-positive control. GAPDH served as a loading control. (B–D) Twelve-week-old male mice were fasted overnight and sacrificed after receiving a single intraperitoneal injection of insulin (0.5 U/kg body weight). The adipose (B), liver (C), and soleus muscle (D) tissues were collected and homogenized, followed by Western blotting analysis using antibodies as indicated. Data are expressed as fold changes relative to the baseline control (time = 0 min) and represented as mean ± SEM, *n* = 4; NS, not significant.
(TIF)

Movie S1 Live imaging of PICK1-containing insulin granules in INS-1E cells. INS-1E cells were transfected with mCherry-PICK1 (red) and phogrin-GFP (green), and live imaging was performed 2 d after transfection (as mentioned in Materials and Methods). PICK1 and phogrin formed co-clusters and moved together in the cytosol of INS-1E cells.
(AVI)

Table S1 Changes of serum melanocortin and lipid profile. Groups of male mice were fasted overnight and sacrificed the next morning for serum collection. Melanocyte-stimulating hormone α (MSH-α), triglycerides, cholesterol, and NEFAs were measured. **p<0.01, Student's *t* test.
(DOC)

## Abbreviations

BAR: Bin/Amphiphysin/Rvs
BFA: Brefeldin A
Co-IP: co-immunoprecipitation
DCV: dense-core vesicle
ELISA: enzyme-linked immunosorbent assay
ER: endoplasmic reticulum
HC: heavy chain
H&E: Haematoxylin and Eosin
IGTT: intraperitoneal glucose tolerance test
ISG: immature secretory granule
ITT: insulin tolerance test
KO: knockout
MSG: mature secretory granule
MSH-α: Melanocyte-stimulating hormone α
NDS: normal donkey serum
NEFAs: nonesterified fatty acids
PDZ: PSD-95/Dlg/ZO1
P-I granules: granules positive for PICK1 and ICA69
PNS: postnuclear supernatant
RER: respiratory exchange rate
TEM: transmitted electron microscope
TGN: Trans Golgi network
WT: wild-type
