# Supplementary figures and images for: PICK1 and ICA69 Control Insulin Granule Trafficking and Their Deficiencies Lead to Impaired Glucose Tolerance
Source: PLoS Biol. 2013 Apr 23;11(4):e1001541. doi: 10.1371/journal.pbio.1001541 (PMC3635858; doi:10.1371/journal.pbio.1001541)

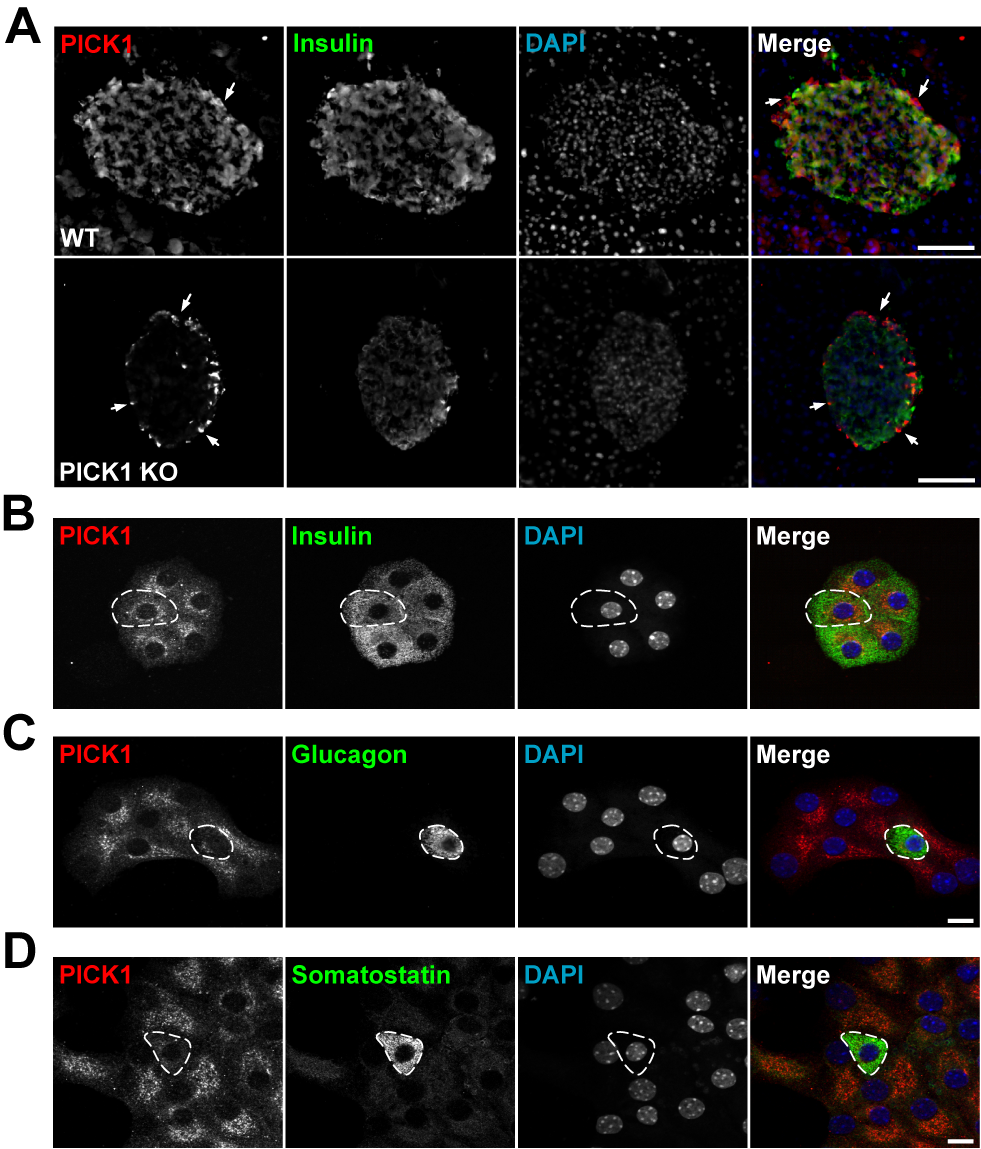

Supplement: Figure S1 — PICK1 is mainly expressed in pancreatic beta cells but not alpha or delta cells. (A) Immunostaining of PICK1 (red) on WT and PICK1 KO pancreatic cryosections. DAPI was used to label nucleus. Guinea pig anti-PICK1 antibody recognizes nonspecific signals at the periphery region of islets (arrows). Scale bar, 100 µm. (B) Double staining of PICK1 (red) and insulin (green) on cultured islet cells. PICK1 is highly expressed in the insulin-positive pancreatic beta cells. Scale bar, 10 µm. (C) Double staining of PICK1 (red) and glucagon (green) on cultured islet cells. PICK1 is weakly expressed in the glucagon-positive pancreatic alpha cells compared with neighboring glucagon-negative cells. Scale bar, 10 µm. (D) Double staining of PICK1 (red) and somatostatin (green) on cultured islet cells. No PICK1 expression could be detected in the somatostatin-positive pancreatic delta cells compared with neighboring somatostatin-negative cells. Scale bar, 10 µm. DAPI was used to label nucleus. (TIF) [file pbio.1001541.s001.tif]

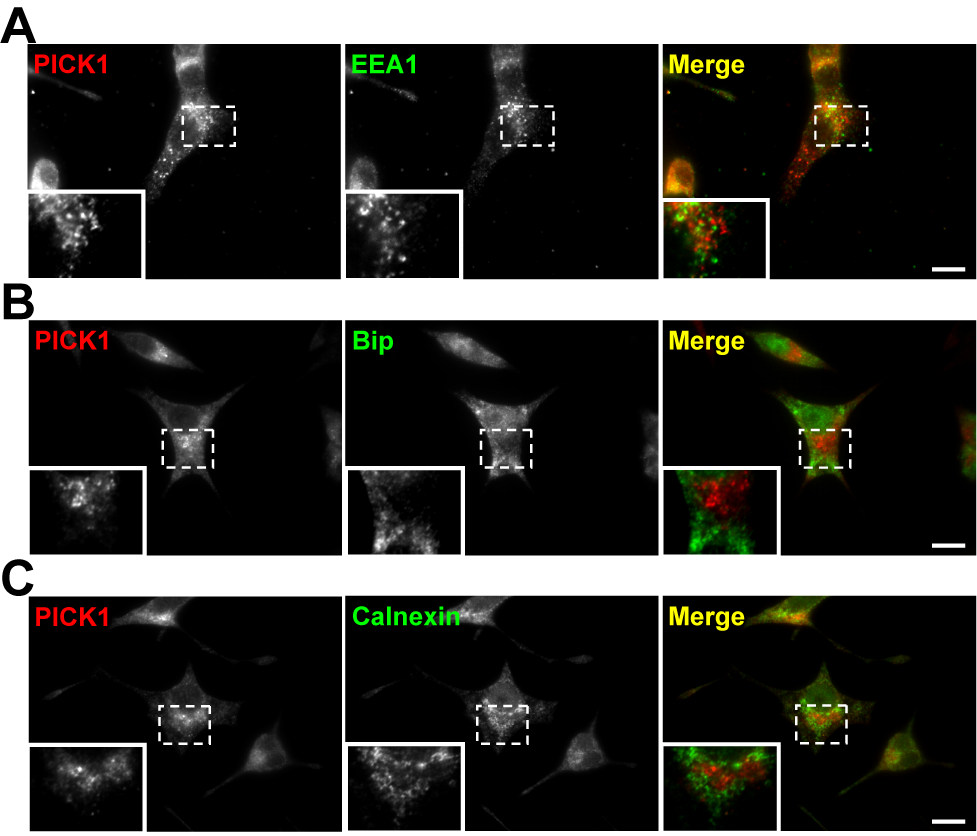

Supplement: Figure S2 — PICK1 vesicles are related to Golgi structures but not early endosomes or ER. Double staining in INS-1E cells for (A) PICK1 (red) and EEA1 (green), (B) PICK1 (red) and Bip (green), and (C) PICK1 (red) and calnexin (green). (A–C) Scale bar, 10 µm. (TIF) [file pbio.1001541.s002.tif]

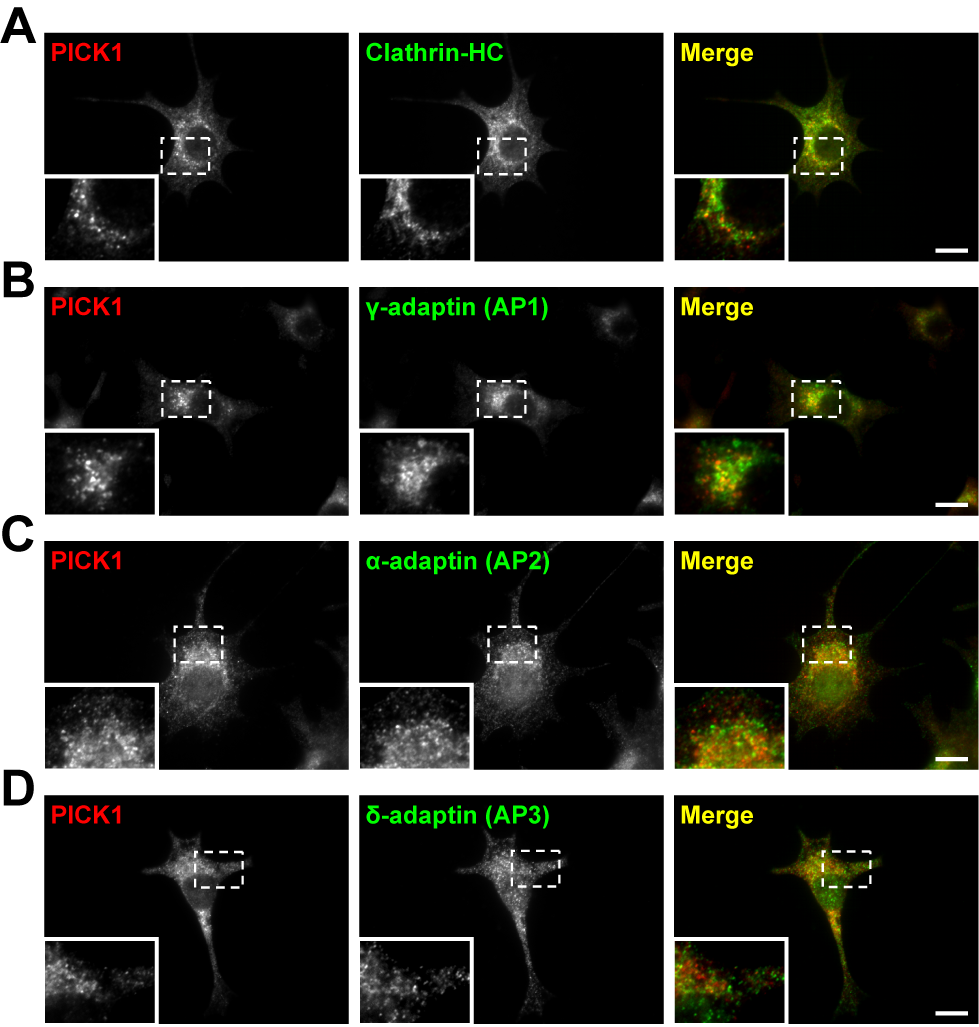

Supplement: Figure S3 — The relationship between PICK1 and clathrin and its adaptors. Double staining in INS-1E cells of (A) PICK1 (red) and Clathrin-HC (green), (B) PICK1 (red) and γ-adaptin (green), (C) PICK1 (red) and α-adaptin (green), and (D) PICK1 (red) and δ-adaptin (green). Scale bar, 10 µm. (TIF) [file pbio.1001541.s003.tif]

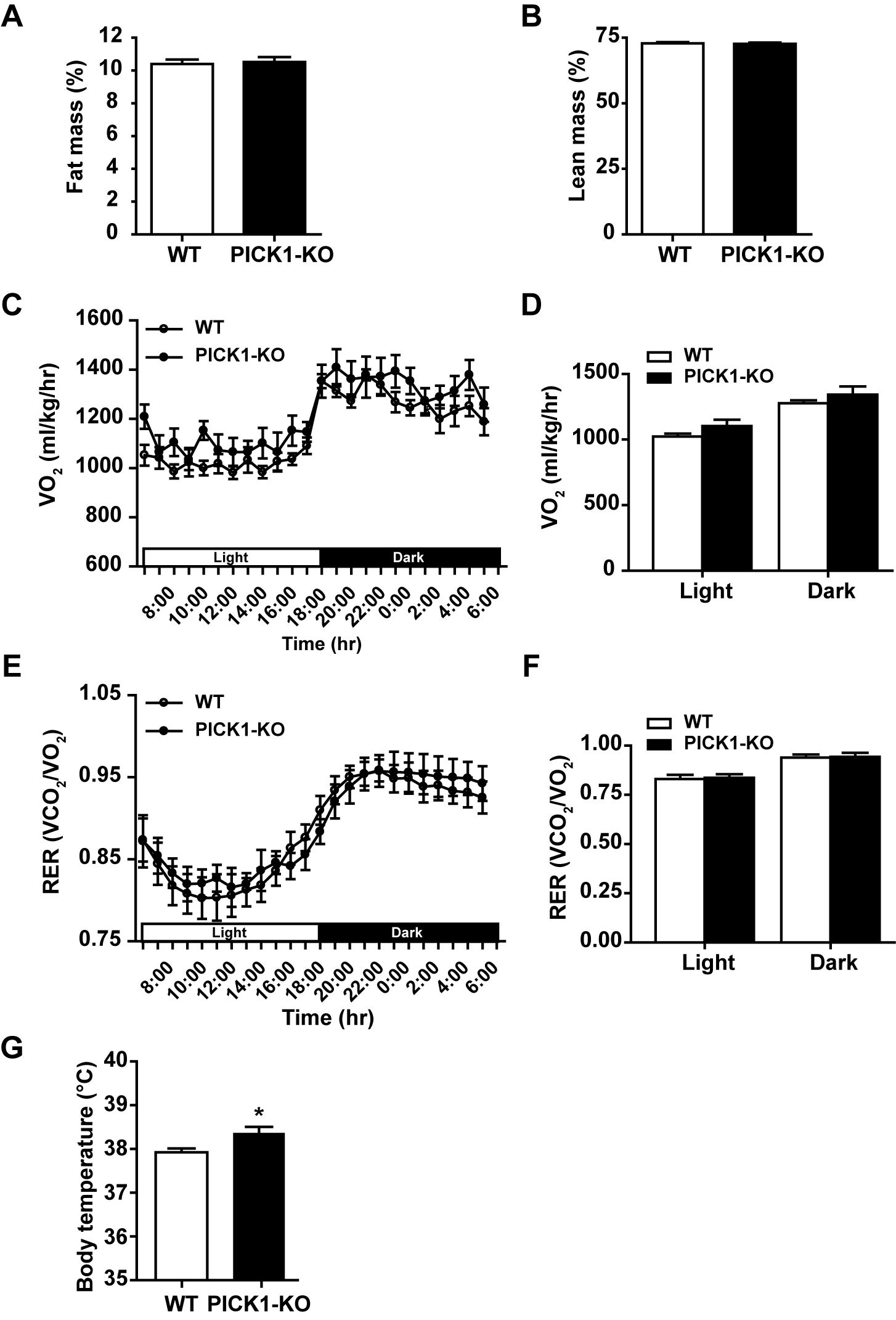

Supplement: Figure S4 — Unaltered body composition and energy expenditure in PICK1 KO mice. (A) Fat mass percentage and (B) lean mass percentage was measured by NMR system (12-wk-old male mice; n = 6). (C–D) Oxygen (O2) consumption. (E–F) Respiratory exchange rate (RER) was measured using metabolic cages for 48 h. (Light, 7 am–7 pm; Dark, 7 pm–7 am; 12-wk-old male mice, n = 6). (G) Body temperature, as measured by anal temperature at 9 am daily (12-wk-old male mice; n = 6). (A–G) Data are represented as mean ± SEM, Student's t test. (TIF) [file pbio.1001541.s004.tif]

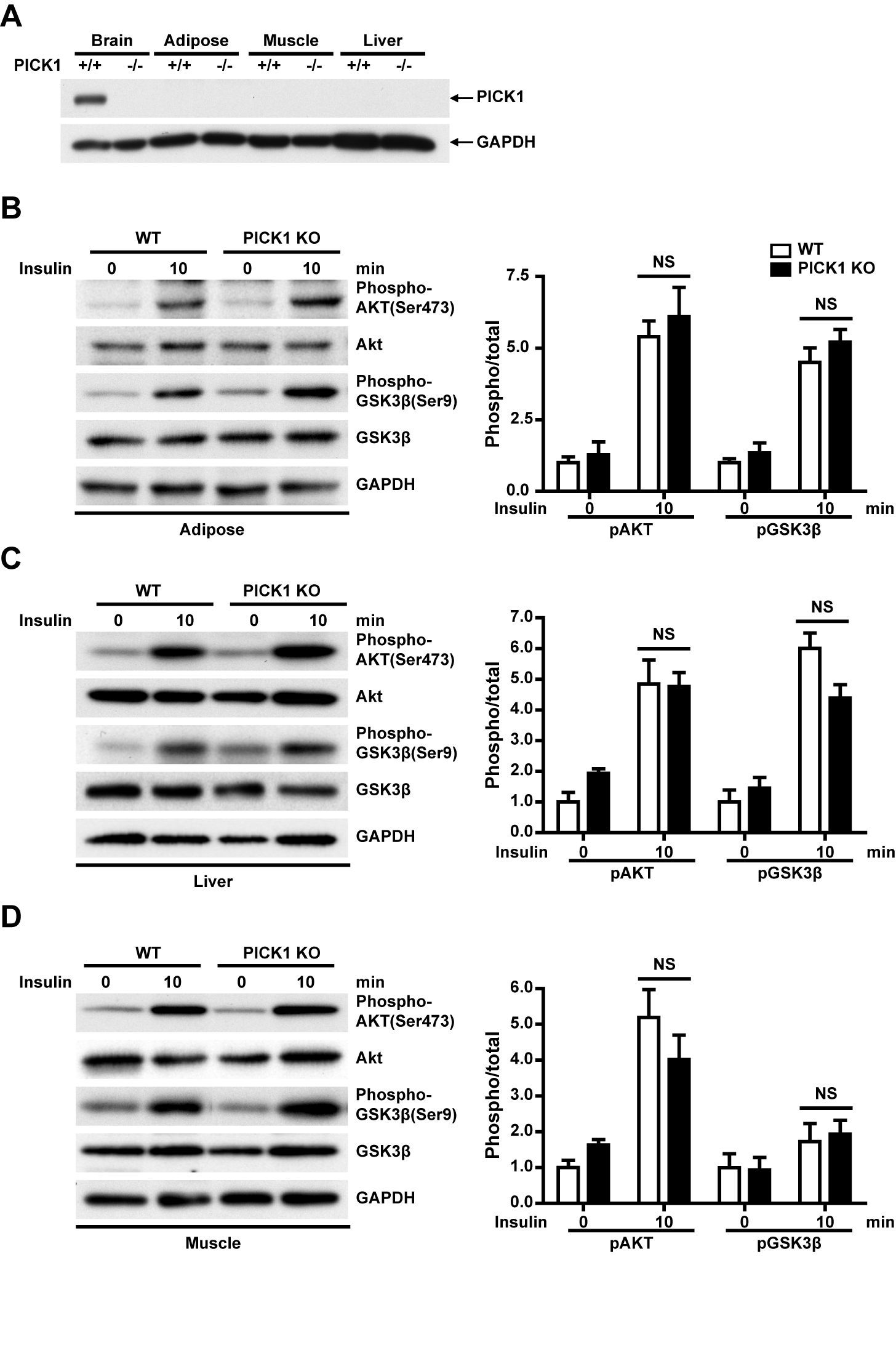

Supplement: Figure S5 — Insulin effects on PICK1 KO target tissues. (A) Western blotting of PICK1 in adipose, muscle, and liver tissue lysates. WT brain tissues were used as a PICK1-positive control. GAPDH served as a loading control. (B–D) Twelve-week-old male mice were fasted overnight and sacrificed after receiving a single intraperitoneal injection of insulin (0.5 U/kg body weight). The adipose (B), liver (C), and soleus muscle (D) tissues were collected and homogenized, followed by Western blotting analysis using antibodies as indicated. Data are expressed as fold changes relative to the baseline control (time = 0 min) and represented as mean ± SEM, n = 4; NS, not significant. (TIF) [file pbio.1001541.s005.tif]
