# Supplementary material for: PICK1 and ICA69 Control Insulin Granule Trafficking and Their Deficiencies Lead to Impaired Glucose Tolerance
Source: PLoS Biol. 2013 Apr 23;11(4):e1001541. doi: 10.1371/journal.pbio.1001541 (PMC3635858; doi:10.1371/journal.pbio.1001541)
Supplement: Table S1 — Changes of serum melanocortin and lipid profile. Groups of male mice were fasted overnight and sacrificed the next morning for serum collection. Melanocyte-stimulating hormone α (MSH-α), triglycerides, cholesterol, and NEFAs were measured. **p<0.01, Student's t test. (DOC) [file pbio.1001541.s007.doc]

**Table S1. Changes of serum melanocortin and lipid profile**

| **Variables** | **WT** | **PICK1-KO** |
| --- | --- | --- |
| MSH-α (µg/µl) | 3.03±0.36 | **2.49±0.32 |
| Triglyceride (mg/dL) | 45.08±2.33 | **36.96±1.60 |
| Cholesterol (mg/dL) | 59.61±3.48 | 62.38±1.82 |
| NEFA (mEq/L) | 0.54±0.05 | 0.48±0.08 |
